# MNT: a new target for AML

**DOI:** 10.1016/j.bneo.2025.100149

**Published:** 2025-08-04

**Authors:** Karla C. Fischer, Veronique Litalien, Sarah T. Diepstraten, Michelle Jahja, Fiona C. Brown, Gemma L. Kelly, Andrew H. Wei, Suzanne Cory

**Affiliations:** 1 Walter and Eliza Hall Institute of Medical Research, Melbourne, VIC, Australia; 2 Department of Medical Biology, The University of Melbourne, Melbourne, VIC, Australia; 3 Department of Clinical Haematology, Peter MacCallum Cancer Centre and Royal Melbourne Hospital, Melbourne, VIC, Australia

## Abstract

Deregulated expression of the transcription factor c-MYC is well established as a primary driver of diverse tumor types. In this study, for the first time to our knowledge, we show that mouse and human myeloid leukemias provoked by oncogenic mixed lineage leukemia (MLL) fusion proteins are dependent on the MYC family member MNT (MAX network transcriptional repressor), which is highly expressed in these AMLs. To investigate the role of MNT, we generated *Mnt-*deletable murine *MLL::AF9* acute myeloid leukemias (AMLs), using the well-studied hemopoietic reconstitution model. *Mnt* deletion provoked the apoptosis of *MLL::AF9* AML cells in vitro and increased apoptosis elicited by the BH3 (BCL-2 homology region 3) mimetic drugs S63845 (MCL-1 (Myeloid cell leukemia-1) specific), ABT-199/Venetoclax (BCL-2 (B-cell lymphoma-2) specific), and A-1331852 (BCL-X_L_ (B-cell lymphoma-extra large) specific). Remarkably, by inducing *Mnt* deletion in vivo in transplanted *MLL::AF9* AMLs, we significantly extended the survival of transplant recipients (*P* < .0001), 50% of which became leukemia free. Using inducible CRISPR/Cas9, we also showed that 3 of 4 human AML cell lines were more potently killed in vitro by BH3 mimetic drugs after *MNT* deletion. Of note, inducing *MNT* deletion in a human *MLL-*rearranged AML cell line transplanted into NSG (NOD SCID Gamma) mice debulked established leukemia and significantly extended the survival of transplant recipients. Taken together with previous studies that demonstrated a critical role for MNT in the development and sustained expansion of B and T lymphomas, our results suggest that a small molecule inhibiting MNT function may be a valuable therapeutic agent for myeloid and lymphoid malignancies.

## Key Points

• Inducing MNT loss in AMLs driven by MLL-fusion proteins extends survival of transplanted mice, indicative of MNT-dependency.
• Inhibiting MNT, a MYC family member, may therefore improve therapy for MLL-driven and perhaps also other AMLs.

## Visual Abstract

**Figure:**
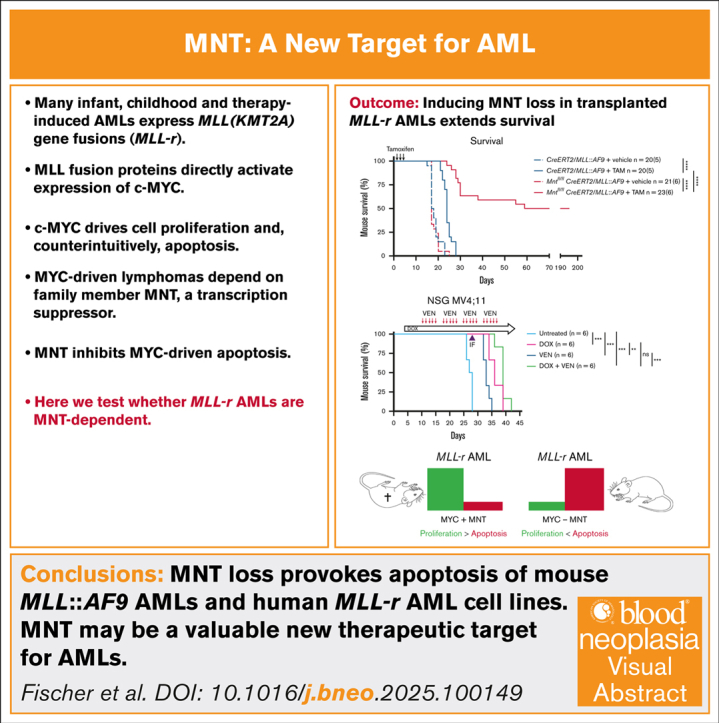

## Introduction

Acute myeloid leukemia (AML) remains a major clinical challenge despite encouraging results from new targeted therapeutic options.^1^^,^^2^ AML can arise from diverse genetic changes.^3^^,^^4^ Among the most common, particularly in infant and childhood AML and in therapy-induced AML, are chromosomal translocations/inversions that involve the gene *MLL* (mixed lineage leukemia), also known as KMT2A (lysine methyl transferase 2A), which is located at chromosome 11q23. *MLL* encodes a large multidomain epigenetic regulator,^5^^,^^6^ and the translocations lead to loss of its C-terminus, which contains a SET (Su(var)3-9, Enhancer-of-zeste, and Trithorax) domain responsible for H3K4 methyltransferase activity. More than 100 fusion partners have been identified, and the consequent MLL fusion proteins differ in potency. Several of the most common (eg, MLL::AF9, MLL::ENL, MLL::AF4, MLL::AF10, and MLL::ELL) constitute a transcriptional activation complex that includes p-TEFb, a cyclin T–dependent kinase that controls elongation by RNA polymerase II, and/or the histone methyltransferase DOT11. Therefore, most *MLL* gene rearrangements (*MLL*-r) lead to overexpression of *MLL* target genes, including *HOXA9* and its co-factor *MEIS1* (reviewed by Schreiner et al^7^).

Importantly, MLL fusion proteins directly activate the expression of c*-*MYC,^8^, ^9^, ^10^ a basic helix-loop-helix leucine zipper (bHLHLZ) transcription factor that controls a host of genes involved in cell growth, cell cycle progression, metabolism, and DNA damage responses.^11^^,^^12^ Counterintuitively, cells that express elevated MYC undergo apoptosis under stress conditions (eg, cytokine or nutrient deprivation),^13^^,^^14^ particularly at high MYC levels.^15^ Although this serves as a critical restraint on MYC-driven neoplastic transformation, the safety mechanism can be overridden by high levels of proteins that enhance cell survival (eg, BCL-2^16^) or loss of proteins that promote cell death (eg, BH3-only protein BIM^17^).

MYC heterodimerizes with MAX (MYC-associated factor X), a related bHLHLZ protein, and together they bind E-box motifs (CACGTG and variants) in regulatory regions of target genes to stimulate their transcription.^18^ MAX also binds several MYC-related transcriptional repressors, including MNT (MAX network transcriptional repressor),^19^ which is widely expressed in mammalian tissues.^19^^,^^20^ MNT/MAX heterodimers suppress transcription by recruiting SIN3/HDAC (switch-independent 3/histone deacetylase) co-regulatory complexes to E-box motifs.^11^^,^^21^^,^^22^

As a MYC antagonist, MNT was expected to be a tumor suppressor. Early studies that showed adenocarcinoma development after conditional *Mnt* deletion in mammary epithelium supported this role.^23^ However, more recently, mouse genetic studies have shown that *Mnt* deletion inhibits B and T lymphoma development by enhancing apoptosis in lymphoid cells that overexpressed MYC.^24^, ^25^, ^26^ Thus, at least in the lymphoid system, MNT actually aids MYC in oncogenesis instead of antagonizing it. It does so by suppressing MYC-driven apoptosis,^23^, ^24^, ^25^ presumably by competing for critical target genes that promote apoptosis, either directly or indirectly.

Because many AMLs express high levels of MYC, which is an important negative prognostic factor,^27^ we wondered whether AMLs, like MYC-driven lymphomas, are dependent on MNT for sustained cell survival and expansion. In this study, we explored the impact of inducing MNT loss in mouse and human AML cells that expressed MLL-fusion proteins and investigated whether MNT loss could enhance the response of these AMLs to BH3-mimetic drugs, which inhibit prosurvival members of the BCL-2 family.^28^^,^^29^

## Materials and methods

The full details are provided in the supplemental Materials and methods.

### Mice

All mice were maintained at the Walter and Eliza Hall Institute (WEHI) on a C57BL/6 background*. Mnt*^*fl/+*^^30^ and *Rosa26*^*CreERT2/+*^^31^ (hereafter referred to as *CreERT2*) mice were crossed to generate *Mnt*^*fl/fl*^ and *Mnt*^*fl/fl*^*/CreERT2* genotypes. Homozygous *Mnt* deletion, reported as being perinatally lethal,^30^ is fatal at around embryonic day 10 (E10) in C57BL/6 mice at WEHI.^25^ *CreERT2* transgene expression enables inducible deletion of floxed *Mnt* alleles by treatment with tamoxifen in vivo or 4-hydroxy tamoxifen (4-OHT) in vitro.^32^

All mice used in this study were bred, housed, and monitored in the Bioservices Facility at WEHI under the supervision of trained veterinarians and in accordance with the WEHI animal ethics committee regulations and the Australian Code for the Care and Use of Animals for Scientific purposes.

### Generation of *Mnt*-deletable murine *MLL::AF9* AMLs

**Figure 1.**
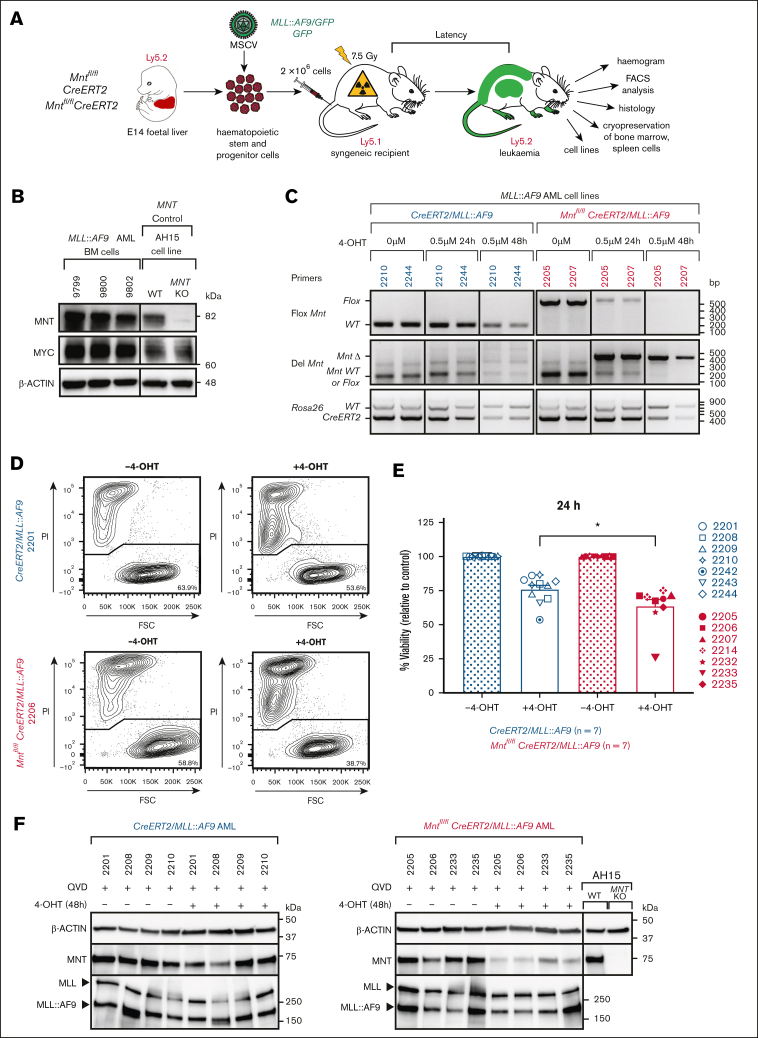
***Mnt* deletion in vitro promotes the death of *MLL::AF9* AML CLs.** (A) Protocol. Fetal liver cells containing hemopoietic stem and progenitor cells (HSPCs) from E14 *Mnt^fl/fl^, CreERT2* or *Mnt*^*fl/fl*^*CreERT2* embryos (C57BL/6-Ly5.2) were infected with either *MLL::AF9/GFP* or control *GFP* MSCV virus and transplanted into sublethally irradiated (7.5 Gy) C57BL/6-Ly5.1 recipients (2 × 10^6^ cells per mouse). Mice that developed leukemia were autopsied, subjected to hemopoietic analysis, and their bone marrow (BM) and spleen cells were cryopreserved. **(**B**)** Robust expression of MNT and MYC proteins in BM of exemplar primary (T0) *MLL::AF9* AML-bearing mice (identified by number), determined by western blot analysis. AH15 is a control Eμ-*Myc* lymphoma CL in which *Mnt* was deleted by CRISPR/Cas9 (C. J. Vandenberg and S. Cory, unpublished data, November 2014). Molecular weight markers (kilodalton) are indicated. The results are representative of independent experiments. (C) Polymerase chain reaction analysis showing the deletion of floxed *Mnt* alleles by *CRE-ERT2* recombinase after treatment with 0.5 μM 4-OHT, indicated by the appearance of a 386 bp *Mnt*Δ fragment and the concomitant loss of a 579 bp *Mnt*^*fl/fl*^ fragment. The results are shown for 2 representative *Mnt*^*fl/fl*^*CreERT2*/*MLL::AF9* AML CLs (2205 and 2207; pink) and 2 control (nondeletable) *CreERT2/MLL::AF9* AML CLs (2210 and 2244; blue). The DNA ladder (base pair) is indicated. (D**)** Flow cytometric analysis of cell viability following treatment with 4-OHT or vehicle (ethanol; –4-OHT) for 24 hours. Representative plots are shown for the indicated *Mnt*^*fl/fl*^*CreERT2*/*MLL::AF9* and control *CreERT2/MLL::AF9* AML CLs. The cells were stained with PI; viable cells are PI-negative. (E) *Mnt* deletion provoked death of the *MLL::AF9* AML cells. Multiple independent *Mnt*^*fl/fl*^*CreERT2/MLL::AF9* (pink) and control *CreERT2/MLL::AF9* (blue) AML CLs were treated in duplicate for 24 hours with 0.5 μM 4-OHT, then washed and resuspended in normal medium and incubated for a further 24 hours before determining the percentage of PI-negative cells by flow cytometry. Two experiments were performed, and a total of 7 *Mnt*^*fl/fl*^*CreERT2/MLL::AF9* and 7 *CreERT2/MLL::AF9* AMLs were tested (see legend); 2 of the former and 3 of the latter were tested in both experiments. The results are presented as mean ± standard error of the mean (SEM) relative to samples incubated without 4-OHT. Significance was determined by 1-way analysis of variance (ANOVA) with Tukey multiple comparison test (∗*P* ≤ .05). (F) MNT loss does not alter the expression of the MLL*::*AF9 fusion protein. A representative western blot analysis is shown for the *CreERT2/MLL::AF9* AML and *Mnt*^*fl/fl*^*CreERT2/MLL::AF9* AML CLs treated with 0.5 μM 4-OHT or vehicle (ethanol) for 48 hours with addition of a pan-caspase inhibitor (QVD-OPH; 25 μM). The expression levels of the MNT, MLL-1, and MLL*::*AF9 proteins are shown with actin as the loading control. FSC, forward light scatter; KO, knockout; PI, propidium iodide; WT, wild type.

Primary AMLs (T0) were generated by *MLL::AF9*/*GFP* retrovirus infection of E14 fetal liver cells from C57BL/6-Ly5.2 mice, followed by transplantation into sublethally irradiated (7.5 Gy) C57BL/6-Ly5.1 recipient mice (Figure 1A), as described previously.^33^^,^^34^

Secondary (T1) AMLs were generated by transplanting spleen cells from primary (T0) AML mice into multiple nonirradiated C57BL/6-Ly5.1 mice of the same sex as the initial E14 embryo; recipients developed secondary (T1) AML within ∼2 weeks.

*MLL::AF*9 AML cell lines (CLs) were generated by culturing bone marrow cells from T0 *MLL::AF*9 AML mice in interleukin-3 as described.^33^^,^^34^ All CLs were regularly tested for mycoplasma (Lonza MycoAlert).

### *Mnt* deletion and drug treatment of *MLL::AF*9 AML CLs

Cells were plated into 96-well flat-bottom plates (5 × 10^4^ cells per well) and incubated for 24 hours in 4-OHT (0.5 μM; catalog no. H7904; Sigma-Aldrich) or vehicle (ethanol), followed by treatment with BH3-mimetic drugs or vehicle for 48 hours. The BH3 mimetics used were S63845 (MCL-1 inhibitor; catalog no. A-6044; Active Biochem), ABT-199/venetoclax (BCL-2 inhibitor; catalog no. A-1231; Active Biochem), and A-1331852 (BCL-X_L_ inhibitor; Guillaume Lessene, WEHI), all dissolved in dimethyl sulfoxide. If required, the pan-caspase inhibitor QVD-OPh (25 μM; catalog no. HY-12305; MedChemExpress) was added 30 minutes before treatment with 4-OHT. Live cells (annexin V–negative propidium iodide–negative) were quantified by flow cytometry.

### *Mnt* deletion in vivo in transplanted *MLL::AF*9 AMLs

Multiple nonirradiated, 8-week-old C57BL/6-Ly5.1 mice were transplanted via tail vein injection with bone marrow cells from a primary (T0) AML mouse (5 × 10^5^ cells per recipient) and treated on days 3, 4, and 5 by oral gavage with tamoxifen (200 mg/kg body weight; catalog no. T5648; Sigma-Aldrich) or vehicle (peanut oil; catalog no. P2144; Sigma-Aldrich). Mice were monitored daily and euthanized humanely at the ethical end point or after 195 days (experimental end point).

For AML treatment with tamoxifen with/without MCL-1 inhibitor S63845, 12 of 24 nonirradiated mice transplanted with 5 × 10^5^ bone marrow cells from T1 AML mice were treated with tamoxifen and 12 of the 24 were treated with vehicle. Subsequently, in each cohort, 6 were injected via the tail vein on days 6 to 10 with S63845 (25 mg/kg body weight; catalog no. 6044; Active Biochem) and 6 with vehicle (2% vitamin E; catalog no. 57668; in Dulbecco's phosphate-buffered saline; Sigma-Aldrich).

### CRISPR/Cas9 deletion of *MNT* in human AML CLs

The human AML CLs used were MV4;11,^35^ THP-1,^36^ MOLM-13,^37^ and OCI-AML3,^38^ authenticated by STR (short tandem repeat) profiling at CellBank Australia. The cells were stably transduced simultaneously with lentiviruses that expressed either FuCas9-Cherry (Addgene Plasmid, catalog no. 70182) or single-guide RNAs (sgRNAs) in doxycycline-inducible FgH1tUTG/GFP (Addgene Plasmid, catalog no. 70183) as described.^39^^,^^40^ To induce sgRNA expression, mCherry^+^GFP^+^ cells were treated with doxycycline hyclate (1 μg/ml; catalog no. D9891; Sigma-Aldrich) for 5 days.

### *MNT* deletion in human AML xenograft mouse model

NOD SCID Gamma (NSG) mice were injected IV with 5 × 10^5^ MV4;11 cells that expressed Cas9 and doxycycline-inducible sgRNAs that targeted human *MNT (MNT2* and *MNT4).* Doxycycline treatment commenced on day 4 after transplantation (600 mg/kg doxycycline hyclate in chow, fed orally ad libitum; catalog no. SF08-026; Specialty Feeds). Treatment with the BCL-2 inhibitor venetoclax (ABT-199) commenced on day 10 (50 mg/kg body weight; every day, except on weekends, by oral gavage, for 4 weeks). Mice were monitored daily and euthanized humanely at the ethical end point. MV4;11 (human CD45^+^) cells were quantified in the bone marrow on day 28 by intrafemoral sampling^41^ and at the experimental end point.

### Statistical analysis

Statistical comparisons were made using a 1-way or 2-way analysis of variance with Tukey multiple comparison test or a 1-way analysis of variance with Dunnett multiple comparison test using Prism software, version 9 (GraphPad, San Diego, CA). Unless otherwise stated, the data are shown as the mean ± standard error of the mean with *P* values of ≤.05 considered statistically significant. Mouse survival curves were plotted in GraphPad Prism, version 9. The curves were compared using the log-rank (Mantel-Cox) test with Bonferroni correction.

## Results

To explore the consequences of MNT loss for *MLL-t* AML, we first focused on the well-studied *MLL::AF9* mouse model^42^^,^^43^ using the *CreERT2* transgene^31^ to conditionally delete floxed *Mnt* alleles in *MLL::AF9* AML cells.

### MNT loss has only a modest impact on normal hemopoiesis

In view of the widespread expression of both *Mnt*^20^ and the *CreERT2* transgene,^31^ before embarking on the AML studies, we investigated whether *Mnt* deletion perturbs normal hemopoiesis. Cohorts of 8 week-old *Mnt*^*fl/fl*^ *CreERT2* mice were treated with tamoxifen to activate the CRE-ERT2 protein^31^ and then analyzed 4 and 8 weeks later, thereby avoiding the reported transient toxicity.^44^ *CreERT2-*mediated *Mnt* deletion was highly efficient as evidenced by polymerase chain reaction and western blot analysis (supplemental Figure 1A-B). The mice remained viable and healthy and only modest hemopoietic changes were observed (supplemental Figure 1C-E; supplemental Table 1). At 4 weeks, the treated mice were mildly leukopenic mainly because of a deficit of lymphocytes and monocytes, but the white blood cell levels returned to normal by 8 weeks. The red blood cell counts were mildly reduced at both 4 and 8 weeks. The major change noted in the bone marrow at 8 weeks was an increase in long-term hemopoietic stem cells (LT-HSC; CD48^–^CD150^+^) and multipotential progenitor 2 cells (MPP2; CD48^+^CD150^+^) in the Lin^-^ Sca1^+^ c-Kit^+^ stem cell compartment. The spleen remained essentially normal. These data indicated that MNT loss did not have a major impact on normal hemopoiesis in the likely time period of the AML study.

### Generation of murine *MLL::AF9* AMLs for conditional *Mnt* deletion

To generate *Mnt*-deletable *MLL::AF9* AMLs, hemopoietic stem/progenitor cells (HSPCs) cells from E14 fetal livers of *Mnt*^*fl/fl*^ *CreERT2* mice were infected with *MLL::AF9*/*GFP* or *GFP* MSCV (Murine Stem Cell Virus) retroviruses and injected into sublethally irradiated (7.5 Gy) recipient mice (Figure 1A). As expected, all mice that were transplanted with *MLL::AF9* virus–infected HSPCs developed typical AML and required ethical euthanasia within 45 days (supplemental Figure 2A), whereas the controls that received *GFP* virus–infected HSPCs remained healthy. The AML-bearing mice presented with leukocytosis, anemia, thrombocytopenia, and splenomegaly (supplemental Figure 2B-E; supplemental Table 2), and their bone marrow was replete with AML cells, which exhibited robust MYC and MNT expression (Figure 1B). These primary AMLs are designated hereafter by the number of the mouse that received transplant, genotype of the donor HSPCs, and virus (eg, 2232 *Mnt*^*fl/fl*^*CreERT2/MLL::AF9* indicates AML that developed in mouse 2232 transplanted with *Mnt*^*fl/fl*^ *CreERT2* fetal liver cells infected with *MLL::AF9/GFP* virus). In all, we generated 6 independent *Mnt*^*fl/fl*^ *CreERT2*/*MLL::AF9* AMLs and, as controls, 5 independent *CreERT2*/*MLL::AF9* AMLs and 2 independent *Mnt*^*fl/fl*^ *MLL::AF9* AMLs.

### *Mnt* deletion reduces the viability of *MLL::AF9* AML cells

To determine the impact of *Mnt* deletion, we first performed in vitro studies. Short-term *MLL::AF9* AML CLs were established by culturing bone marrow cells from primary AML mice in interleukin-3–containing medium.^33^^,^^34^ Polymerase chain reaction tests indicated that *Mnt* deletion using 0.5 μM 4-OHT was largely complete by 24 hours (Figure 1C**)**. Multiple independent CLs (n = 7) were treated with 4-OHT or vehicle, and cell viability was determined by flow cytometry after 24 hours (Figure 1D**)**. The viability of control (*Mnt*^+/+^) *CreERT2/MLL::AF9* CLs (blue) decreased after exposure to 4-OHT, as previously noted,^45^ probably because of off-target CRE-mediated toxicity. Nevertheless, the viability of *Mnt*^*fl/fl*^ *CreERT2/MLL::AF9* AML CLs (pink) was significantly lower again (*P* = .0170; Figure 1E) because of the *Mnt* deletion. Importantly, MNT loss did not alter the level of expression of the driver fusion protein MLL::AF9 (Figure 1F).

### *Mnt* deletion enhances sensitivity of *MLL::AF9* AML CLs to BH3 mimetic drugs

**Figure 2.**
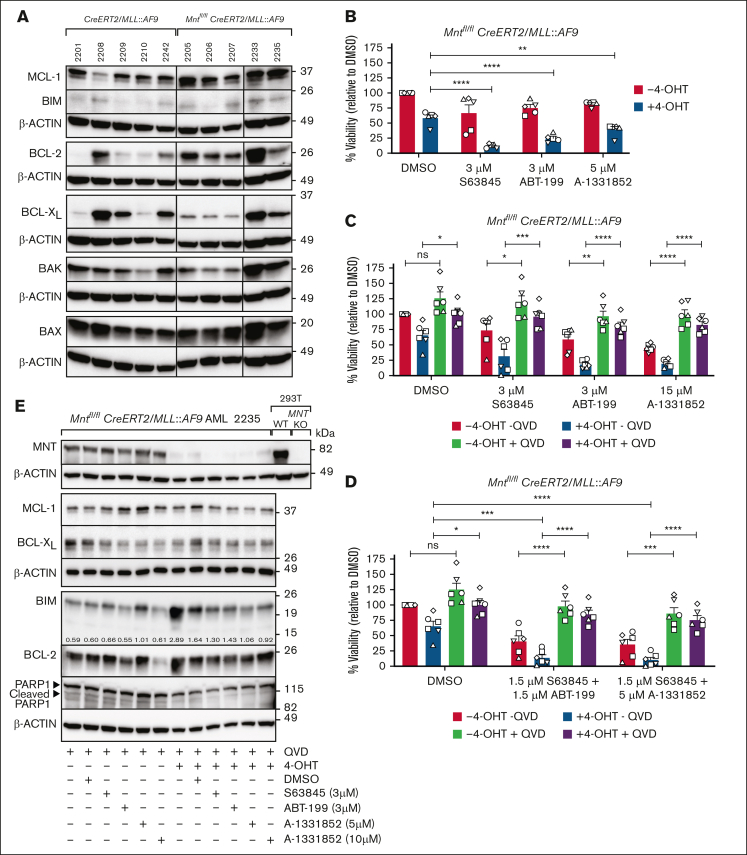
***Mnt* deletion increases the susceptibility of *MLL::AF9* AML CLs to BH3 mimetic drugs.** (A) Expression of BCL-2 family members in primary *Mnt*^*fl/fl*^*CreERT2/MLL::AF9* AML CLs and control *CreERT2/MLL::AF9* AML CLs. MCL-1, BCL-2, and BCL-X_L_ promote cell survival. BAX, BAK, and BH3-only protein promote apoptosis. The western blots used β-actin as a loading control. Molecular weight markers are indicated (kilodalton). The results are representative of independent experiments. (B) *Mnt* deletion enhances the killing of AML cells by BH3 mimetic drugs. Short-term AML CLs established from 5 independent *Mnt*^*fl/fl*^*CreERT2/MLL::AF9* AML tumors (2205, circle; 2206, square; 2207, triangle up; 2233, triangle down; 2235, diamond) were treated for 24 hours in triplicate with 0.5 μM 4-OHT or vehicle (ethanol), followed by treatment with the BH3 mimetic drugs S63845 (MCL-1 inhibitor), ABT-199/venetoclax (VEN; BCL-2 inhibitor), or A-1331852 (BCL-X_L_ inhibitor) for a further 24 hours at the indicated concentrations, or with vehicle (DMSO), in the presence of 4-OHT. The BH3 mimetic drug concentrations used were based on predetermined 50% inhibitory concentration (IC50) values (data not shown). Live cells were quantified using annexin V–negative PI-negative staining with flow cytometry (supplemental Figure 3), and cell viability is shown normalized to treatment with DMSO. The results are from 2 independent experiments; mean ± standard error of mean (SEM); ∗∗*P* ≤ .01; ∗∗∗∗*P* ≤ .0001 as determined by 1-way ANOVA with Dunnett multiple comparison test. (C) *MLL::AF9* AML cell death after *Mnt* loss and treatment with BH3 mimetic drugs occurs by apoptosis. *Mnt*^*fl/fl*^*CreERT2/MLL::AF9* AML CLs used in panel B were treated in triplicate with 4-OHT or vehicle (ethanol) for 24 hours, with or without the addition of a pan-caspase inhibitor (QVD-OPH; 25 μM), and then treated for 24 hours with BH3 mimetic drugs or vehicle (DMSO). Viable cells (annexin V–negative PI-negative) were quantified by flow cytometry. Results are from 3 independent experiments with 1 CL (2206) analyzed twice; mean ± SEM; ns, *P* > .05; ∗*P* ≤ .05; ∗∗*P* ≤ .01; ∗∗∗*P* ≤ .001; ∗∗∗∗*P* ≤ .0001 as determined by 1-way ANOVA with Tukey multiple comparison test. (D) Apoptosis is enhanced by combining *Mnt* deletion with synergistic concentrations of BH3 mimetic drugs. The *Mnt*^*fl/fl*^*CreERT2/MLL::AF9* AML CLs described in panel B were treated in triplicate with 0.5 μM 4-OHT or vehicle (ethanol), with or without QVD-OPH (25 μM), for 24 hours and then treated for 24 hours with either 1.5 μM S63845 + 1.5 μM ABT-199 or 1.5 μM S63845 + 5 μM A-1331852 or carrier alone (DMSO). Viable cells (Annexin V-negative PI-negative) were identified by flow cytometry. Results are from 3 independent experiments with 1 CL (2206) analyzed twice. The data are presented as mean ± SEM; ns, *P* > .05; ∗*P* ≤ .05; ∗∗∗*P* ≤ .001; ∗∗∗∗*P* ≤ .0001 as determined by 2-way ANOVA with Tukey multiple comparison test. (E) Proapoptotic BIM is elevated in MNT-deficient AML cells. Western blot analysis of 2235 *Mnt*^*fl/fl*^*CreERT2/MLL::AF9* AML CL treated as in panel C, showing the protein levels of MNT, BCL-2 family members, and PARP1, with actin as the loading control. The indicated ratios show the BIM level in comparison with that of actin on the same blot, determined by densitometric analysis using ImageJ software. Cleavage of PARP1, a marker of apoptosis, is inhibited in the presence of QVD-OPH. DMSO, dimethyl sulfoxide; KO, knockout; ns, not significant; PARP, poly ADP ribose polymerase; WT, wild type.

The BCL-2 family of prosurvival and proapoptosis proteins regulates the mitochondrial cell death pathway.^28^ *MLL::AF9* AML CLs express prosurvival BCL-2, MCL-1, and BCL-X_L_ (Anstee et al^34^; Figure 2A), and MCL-1 is essential for the development and sustained growth of AML driven by *MLL* fusion genes.^45^ Recently, BH3 mimetic drugs that inhibit individual prosurvival family members have been developed to promote the apoptosis of cancer cells,^46^ and the BCL-2 inhibitor venetoclax is being used in combination therapy to treat AML.^47^To explore whether MNT loss could sensitize the AML cells to BH3 mimetic drugs, 5 independent *Mnt*^*fl/fl*^ *CreERT2/MLL::AF9* AML CLs were treated for 24 hours with 4-OHT or vehicle (ethanol) and then for another 24 hours with S63845 (MCL-1 inhibitor), ABT-199/venetoclax (BCL-2 inhibitor), A-1331852 (BCL-X_L_ inhibitor), or vehicle (dimethyl sulfoxide) in the presence of 4-OHT. Cell death was significantly increased by combining *Mnt* loss (+4-OHT) with exposure to each of the BH3 mimetic drugs (Figure 2B). The inclusion of the pan-caspase inhibitor QVD-OPH (Quinoline-Val-Asp-Difluorophenoxymethylketone) in the medium enhanced cell survival, indicating that cell death provoked by *Mnt* loss alone, or in combination with BH3 mimetic drugs, was by apoptosis (Figure 2C). Combining BH3 mimetics with different specificities was more effective than using a higher concentration of either alone, and *Mnt* loss again augmented killing, which was dependent on caspase activity (Figure 2D). Western blot analysis of QVD-OPH–treated *Mnt*^*fl/fl*^*CreERT2/MLL::AF9* AML cells derived from mouse 2235 (Figure 2E) suggested that the increased efficacy of BH3 mimetic drugs after *Mnt* deletion was because of a further elevation of the BH3-only protein BIM (Bcl-2 Interacting Mediator of cell death), a proapoptotic member of the BCL-2 family.^48^ No consistent changes were observed in the level of the prosurvival proteins MCL-1, BCL-X_L_, or BCL-2.

### Impact of *Mnt* deletion in transplanted *MLL::AF9* AMLs

**Figure 3.**
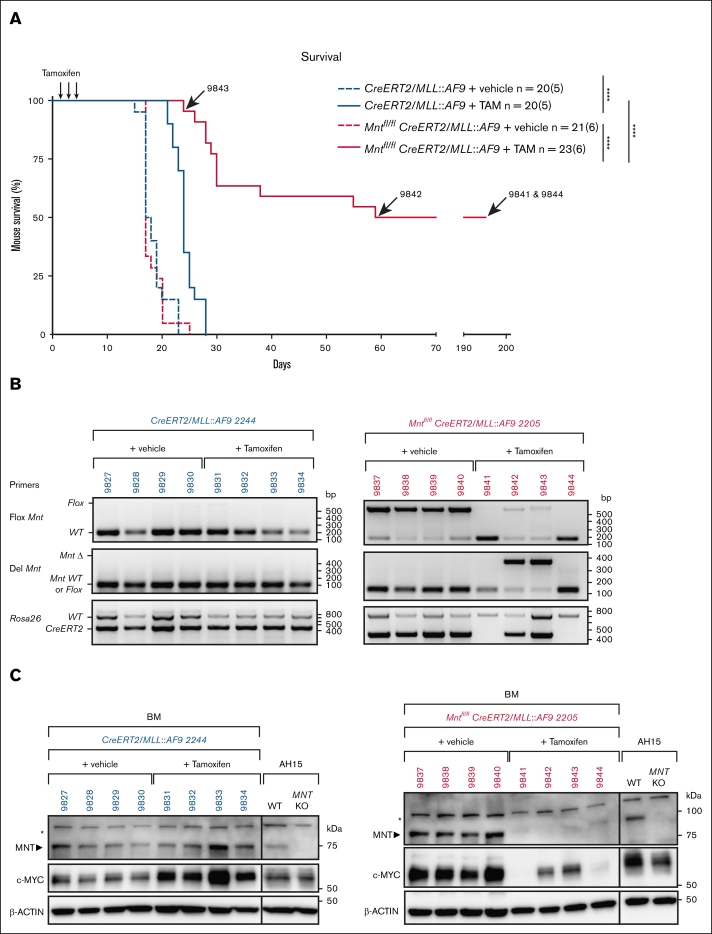
***Mnt* deletion enhances the survival of mice transplanted with primary *MLL::AF9* AML cells.** (A) Kaplan-Meier survival analysis of mice transplanted with primary (T0) *MLL::AF9* AMLs of the indicated genotypes. Eight mice were transplanted IV with 5 ×10^5^ BM cells from each primary (T0) AML mouse on day 0 and treated by oral gavage with TAM (200 mg/kg body weight) or vehicle on days 3, 4, and 5. TAM-treated mice transplanted with *Mnt*^*fl/fl*^*CreERT2/MLL::AF9* AML cells showed significantly delayed morbidity (pink curve; median survival, 57 days) when compared with the TAM-treated control mice transplanted with *CreERT2/MLL::AF9* AML cells (blue curve; median survival, 24 days; supplemental Table 3). The arrows indicate the lifespan of mice with AMLs 9843, 9842, 9841, 9844. n = number of recipient mice; number in brackets indicates the number of independently derived *MLL::AF9* AMLs transplanted. Statistical significance was determined using a log-rank (Mantle-Cox) test with Bonferroni correction (∗∗∗∗*P* < .0001). (B-C) *Mnt* deletion was assessed by polymerase chain reaction (B) and western blot (C) analysis of the BM cells obtained from individual transplant recipients at autopsy. Results are shown for 2 representative primary AMLs, *CreERT2/MLL::AF9* 2244 (blue) and *Mnt*^*fl/fl*^*CreERT2/MLL::AF9* 2205 (pink); individual transplant recipient mice are identified. The DNA ladder (base pair) and marker protein sizes (kilodalton) are indicated. Of note, in panel B, no AML cells were detectable in the BM of cured mice (9841 and 9844), as shown by the absence of the 450 bp CreERT2-specific DNA fragment. Panel C showing the MNT and MYC protein levels with actin as the loading control. Both MYC and MNT are undetectable in the BM of cured’ mice (9841 and 9844), as is the case for normal BM cells (not shown). Asterisks indicate a nonspecific band; AH15 is a control Eμ-*Myc* lymphoma CL in which *Mnt* was deleted by CRISPR/Cas9. KO, knockout; TAM, tamoxifen; WT, wild type.

We next tested the impact of *Mnt* deletion in transplanted *MLL::AF9* AMLs (Figure 3A). Bone marrow cells from each primary (T0) AML mouse were injected IV into a cohort of 8 nonirradiated C57BL6 mice, 4 of which received tamoxifen (200 mg/kg) and 4 of which received only vehicle (negative control). A total of 13 independent primary AMLs were transplanted in 6 different experiments, each of which included both *Mnt*-deletable and control *Mnt* nondeletable genotypes. The AML cell death induced by MNT loss would be expected to improve the survival of transplant recipients.

Regardless of the AML genotype, the median survival of transplant recipients that were treated with vehicle was 17 days (pink and blue broken lines in Figure 3A). Control *CreERT2/MLL::AF9* AML recipients that were treated with tamoxifen (blue solid line) survived longer (∼24 days), probably because of the impact of tamoxifen and/or off-target CRE activity. However, of note, the survival of *Mnt*^*fl/fl*^ *CreERT2/MLL::AF9* AML recipients treated with tamoxifen (solid pink line) was considerably longer (median, 57 days; *P* < .0001).

Remarkably, 11 of the 22 tamoxifen-treated *Mnt*^*fl/fl*^ *CreERT2/MLL::AF9* AML recipients (2/4 transplanted with 2205, 2/3 with 2206, 4/4 with 2235, and 3/3 with 2233) seemed to be cured (supplemental Table 3). Analysis of these survivors 195 days after transplantation revealed that their blood profiles had returned to normal and that the AML cells (ie, *CreERT2*^+^, *Mnt*Δ^+^, MYC protein^hi^) were largely undetectable in the bone marrow, as exemplified for mouse 9841 and mouse 9844 in Figure 3B-C.

The other 11 tamoxifen-treated *Mnt*^*fl/fl*^ *CreERT2/MLL::AF9* AML recipients developed typical leukemia, with a mean survival of 30 days (supplemental Table 3). When autopsied, these mice had high white blood cell counts and low platelet counts, and their bone marrow and spleen was dominated by *Mnt*-deleted AML cells that expressed high levels of MYC protein (eg, 9842, 9843 in Figure 3B-C). We infer that, despite experiencing some initial survival benefit, these mice were eventually overwhelmed by clone(s) bearing mutations that conferred resistance to MNT loss. We have not yet identified these lesion(s) but have ruled out loss of proapoptotic BIM and, for 2 tumors, loss of proapoptotic BAX or BAK (not shown).

Taken together, these results suggest that MNT loss very significantly disadvantages *MLL::AF9* AML cells in vivo, presumably by enhancing cell death, but mutations that confer resistance can emerge.

### Testing *Mnt* deletion combined with MCL-1 inhibitor S63845

**Figure 4.**
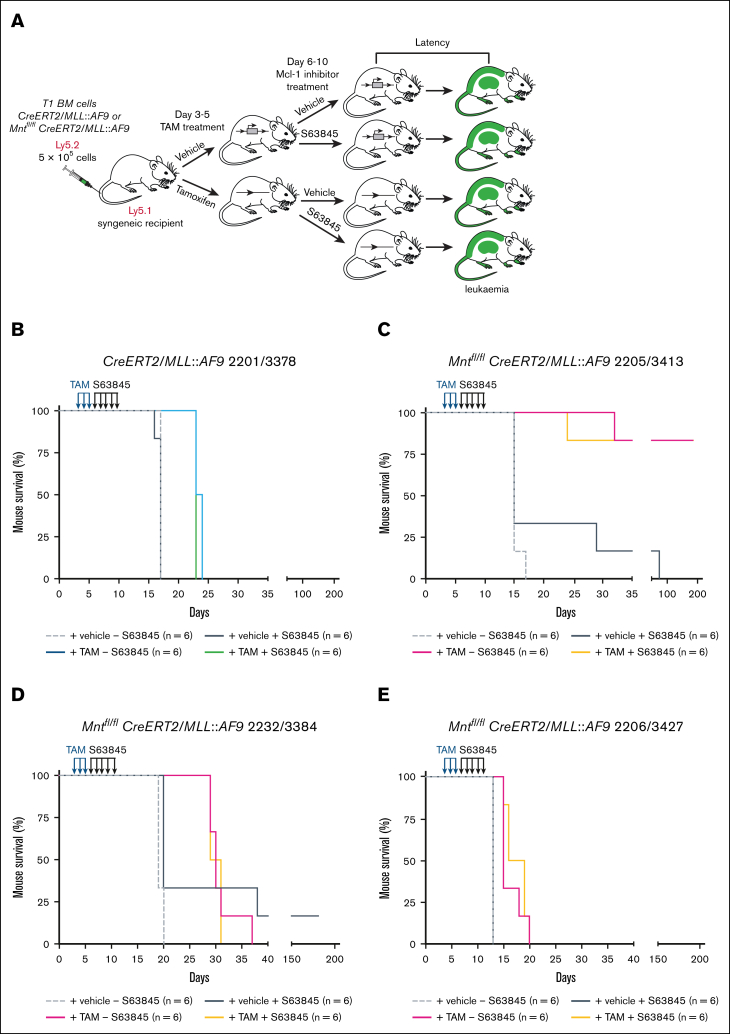
**Treatment with MCL-1 inhibitor S63845 following *Mnt* deletion does not further enhance the survival of mice transplanted with T1 *MLL::AF9* AMLs.** (A) Treatment protocol. BM cells (Ly5.2) cryopreserved from sick secondary (T1) *Mnt^fl/fl^ CreERT2/MLL::AF9* or *CreERT2/MLL-AF9* mice were transplanted on day 0 into 8-week-old nonirradiated C57BL/6-Ly5.1 syngeneic recipient mice (5 × 10^5^ cells into each of 24 recipients). A total of 12 recipients were treated on days 3, 4, and 5 with TAM (200 mg/kg), and 12 were treated with vehicle (peanut oil). Then, starting on day 6, half of the mice in each treatment arm were injected on 5 consecutive days with the MCL-1 inhibitor S63845 (25 mg/kg) and half were injected with vehicle. The mice were monitored daily for disease symptoms and euthanized humanely when they showed signs of AML-induced stress or at the experiment end point (day 195). (B-E) Kaplan-Meier survival analysis of mice transplanted with T1 *MLL::AF9* AMLs of the indicated genotypes and treatments (for T1 AML nomenclature, see Materials and methods and supplemental Table 4).

In view of the MCL-1 dependency of MLL fusion AMLs,^45^ we wondered whether combining *Mnt* deletion with S63845 treatment would be more efficacious than either alone. For these experiments, because stocks of T0 AML bone marrow cells had been depleted, we used bone marrow cells from secondary (T1) AML mice, generated by transplanting cryopreserved spleen cells from T0 AML mice. The treatment protocol is outlined in Figure 4A and outcomes are summarized in Figure 4B-E and supplemental Table 4.

*Mnt* deletion alone (tamoxifen treatment; solid pink curve) prolonged survival of the recipient mice, but the extent varied for individual T1 AMLs. The impressive effect for T1 3413 (Figure 4C; mean survival, >195 days), which derived from T0 2205 (hence designated 2205/3413), mirrored that of T0 2205 (mean survival, 127 days; supplemental Table 3). T1 2232/3384 also responded to *Mnt* deletion in a similar manner as T0 2232 (Figure 4D; mean survival, 30 days vs 28.5 days, respectively). However, T1 2206/3427 (Figure 4E) was far less responsive than T0 2206 (mean survival, 16.5 days vs 85 days), presumably because further mutation had occurred during the expansion in vivo before testing.

Although treatment with S63845 alone (compare solid black and broken gray curves) prolonged the survival of some T1 *MLL::AF9* AML-transplanted mice (outcome for 2205/3413, 2232/3384; Figure 4C-D), it had no impact on others (T1 2201/3378 and T1 2206/3427; Figure 4B,E). Furthermore, it did not provide any further survival benefit to mice that had first been treated with tamoxifen to delete *Mnt* (compare pink and orange curves; Figure 4C-E).

### Does MNT loss enhance the vulnerability of human AML CLs?

**Figure 5.**
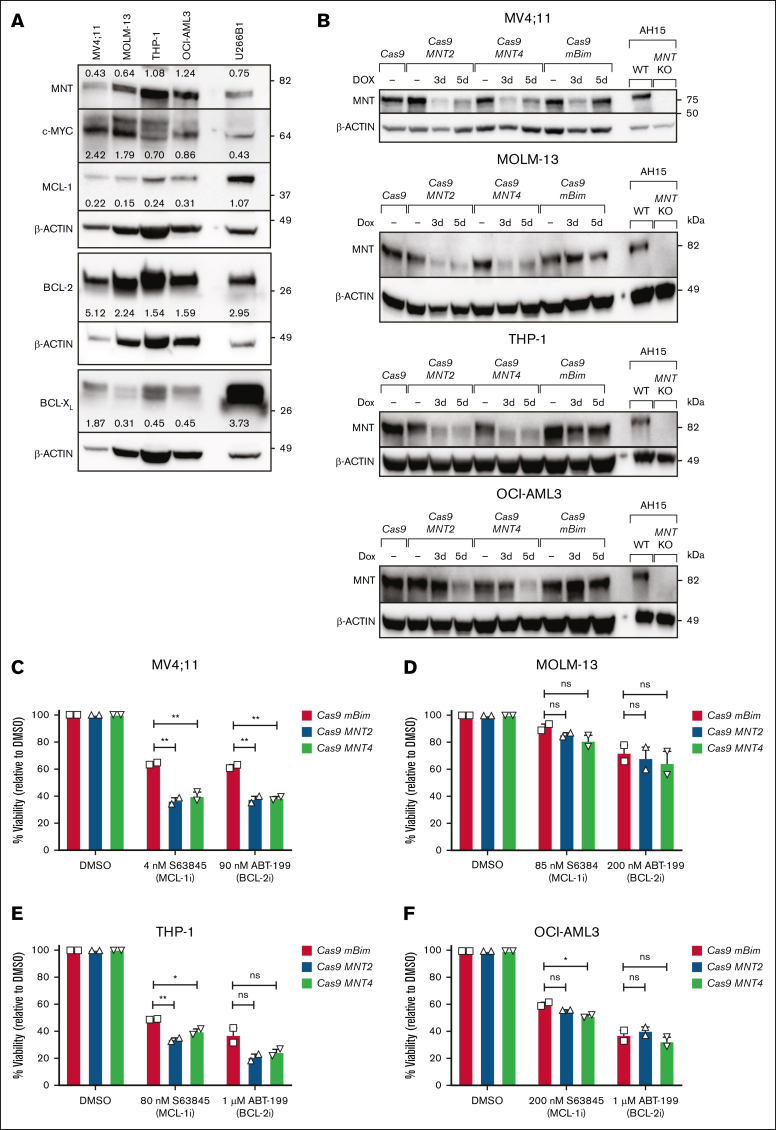
**Impact of CRISPR/Cas9-mediated *MNT* deletion on the sensitivity of human AML CLs to BH3 mimetic drugs.** (A) The expression of MYC, MNT, and the indicated BCL-2 protein family members in the human AML CLs MV4;11, MOLM-13, THP-1, OCI-AML3 and, as a positive control, the multiple myeloma CL U266B1, as determined by western blot. The protein levels were quantified in each blot relative to the actin loading control by densitometric analysis using ImageJ software; the values are indicated. (B) Western blot analysis of CRISPR/Cas9-mediated MNT loss in human AML CLs. MV4;11, MOLM-13, THP-1, and OCI-AML3 cells were infected with 2 lentiviruses, 1 carrying Cas9 and the mCherry marker and the other carrying an sgRNA and GFP marker. Double-positive (mCherry^+^GFP^+^) cells were collected by flow cytometry and treated with DOX for 3 or 5 days to induce expression of the sgRNAs that target *MNT* (*MNT*2, *MNT*4) or mouse *Bim (mBim).* AH15 is a control Eμ-*Myc* lymphoma CL in which *Mnt* was deleted by CRISPR/Cas9. (C-F) The impact of MNT loss on the sensitivity to BH3 mimetics. MV4;11, MOLM-13, THP-1, and OCI-AML3 cells infected with Cas9 and sgRNA lentiviruses were treated with DOX for 3 days, followed by treatment with the BH3 mimetic drugs S63845 (MCL-1i) or ABT-199/VEN (BCL-2i) in the presence of DOX for 48 hours. BH3 mimetic drug concentrations used were based on predetermined individual IC_50_ values. Live cells were identified as annexin V–negative/PI-negative by flow cytometry. The data are shown normalized to the DMSO-treated control samples, and significance is indicated relative to the control sgRNA *mBim*. All data are presented as mean ± SEM of 2 independent experiments. A 1-way ANOVA with Dunnett multiple comparison test was used to determine statistical significance (ns, *P* > .05; ∗*P* ≤ .05; ∗∗*P* ≤ .01). BCL-2i, BCL-2 inhibitor; DOX, doxycycline; KO, knockout; MCL-1i, MCL-1 inhibitor; WT, wild type.

To extend our studies to human AML, we investigated 4 patient-derived human AML CLs, 3 of which had MLL fusion genes (MV4;11; *MLL::AF4*,^35^ MOLM-13; *MLL::AF9*,^37^ and THP-1; *MLL::AF9*),^36^ and the fourth (OCI-AML3) had NPM1 and DNMT3A mutations.^38^ Each of these long-established lines expresses MYC and MNT, as well as varying levels of the prosurvival proteins BCL-2, BCL-X_L_, and MCL-1 (Figure 5A).

To perform the CRISPR/Cas9 deletion, we used a dual lentiviral vector system^39^^,^^40^ to introduce *CAS9* and sgRNAs. Two independent sgRNAs that targeted human *MNT* (*MNT*2, *MNT*4) were tested, as well as an sgRNA for mouse *Bim* as a nontargeting control. After transduction, viable cells that expressed high levels of both mCherry (CAS9^+^) and GFP (Green fluorescent protein) (sgRNA^+^) were isolated by fluorescence-activated cell sorting and treated with doxycycline to induce sgRNA expression. Western blot analysis indicated reduced MNT protein levels after 3 to 5 days (Figure 5B).

To assess whether MNT loss increased the sensitivity to BH3 mimetic drugs, we treated the pooled populations of mCherry^hi^GFP^hi^ cells with doxycycline for 3 days, followed by treatment with the BH3 mimetic drugs S63845 (MCL-1 inhibitor) or ABT-199/venetoclax (BCL-2 inhibitor) in the presence of doxycycline for a further 2 days. The BH3-mimetic concentrations used were based on predetermined 50% inhibitory concentration values. Figure 5C-F shows that MNT protein depletion enhanced the response of MV4;11, THP-1, and OCI-AML3 cells to S63845 and, in the case of MV4;11, also to ABT-199 (venetoclax). However, the sensitivity of MOLM-13 cells was minimally affected.

### MNT loss induced in MV4;11 AML mouse xenografts significantly enhanced the survival of transplant recipients

**Figure 6.**
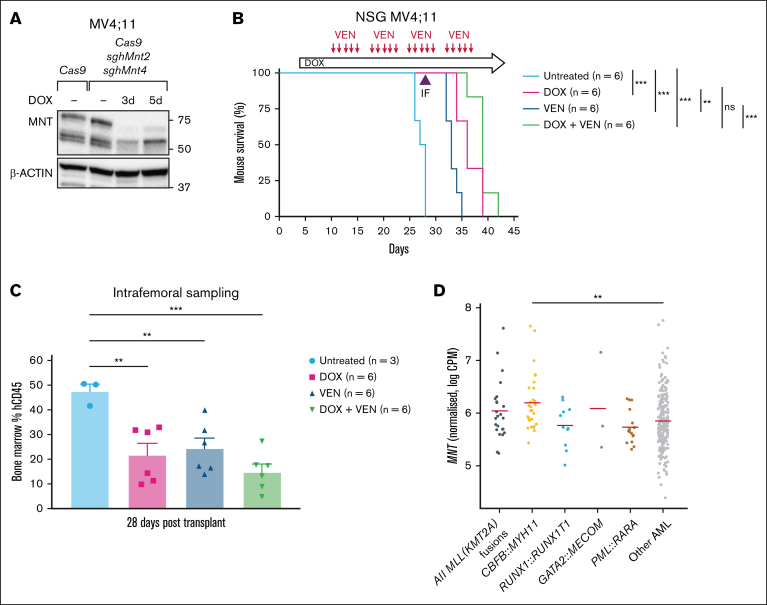
***MNT* deletion enhances the survival of NSG mice engrafted with MV4;11 human AML cells.** (A) Western blot showing the in vitro *MNT* deletion after DOX induction of sgRNA expression in the batch of MV4;11 human AML cells used for transplantation into NSG mice. (B) DOX-induced MNT loss in MV4;11 human AML cells in vivo enhanced the survival of NSG mice that received transplants. Transplant recipients treated with DOX (pink curve) survived significantly longer than the untreated mice (compare pink and pale blue curves) and those treated with VEN (compare pink and dark blue curves). Combining VEN with MNT loss (green curve) did not statistically improve the survival when compared with MNT loss alone (pink curve). NSG mice were transplanted with 5 × 10^5^ MV4;11 human AML cells that coexpressed Cas9 and 2 independent DOX-inducible sgRNAs that targeted human *MNT (MNT2* and *MNT4)*. Recipient mice were either left untreated, treated with DOX to delete *MNT*, treated with the BH3 mimetic drug VEN to inhibit BCL-2, or treated with both DOX and VEN (6 mice per treatment arm). Dosing with DOX to induce expression of *MNT* sgRNAs commenced on day 4 after transplantation (600 mg/kg DOX hyclate in base rodent chow, fed orally ad libitum). Dosing with VEN (50 mg/kg, by oral gavage every day except weekends, for 4 weeks) commenced on day 10 after transplantation. Statistical significance was determined using a log-rank (Mantle-Cox) test with Bonferroni correction (ns, *P* > .05; ∗∗*P* ≤ .01; ∗∗∗*P* ≤ .001). (C) *MNT* deletion and VEN treatment significantly reduced the tumor burden in the BM of mice transplanted with MV4;11 cells. Flow cytometric analysis of hCD45^+^ cells in BM isolated by intrafemoral sampling of mice in panel B, 28 days after transplantation. Reduction of AML cells was most efficient for the combination of *MNT* deletion plus VEN treatment (compare green to pale blue column); the differences between the various treatment groups did not reach statistical significance. The data are presented as the mean ± SEM of 3 to 6 mice per treatment arm. A 1-way ANOVA with Dunnett multiple comparison test was used to determine statistical significance (∗∗*P* ≤ .01; ∗∗∗ *P* ≤ .001). (D) A comparison of MNT messenger RNA expression in AMLs that had *MLL* (*KMT2A*) or other indicated fusion genes, other types of AMLs, and CD34^+^ HSCs from healthy individuals, determined by the analysis of BEAT-AML 1.0 (accessed on 16 May 2025; https://registry.opendata.aws/beataml). hCD45, human CD45; sgh, short guide human.

Next, we turned to a xenograft model to test the impact of MNT loss in vivo in comparison with the outcome of treatment with venetoclax (ABT-199), which is approved for treating certain patients with AML.^49^ For this trial, MV4;11 human AML cells transduced with Cas9 and *MNT*2 sgRNA (Figure 5B) were additionally transduced with the *MNT*4 sgRNA vector. In vitro testing of mCherry^hi^GFP^hi^ cells to be used for the xenograft showed highly effective MNT loss after 3 days of doxycycline treatment (Figure 6A). Of the 24 transplanted NSG mice, 6 were left untreated and the others (3 cohorts of 6 mice each) were treated with doxycycline alone (provided in chow throughout the experiment, starting on day 4), venetoclax alone, or doxycycline plus venetoclax.

Notably, MNT loss induced by doxycycline treatment significantly prolonged the survival of transplant recipients when compared with the untreated control mice (compare pink and pale blue curves; *P* < .001). Indeed, survival was better with MNT loss than with venetoclax treatment (compare pink and dark blue curves; *P* < .01). Although combining venetoclax with MNT loss (green curve) did not statistically improve the survival when compared with MNT loss alone (pink curve), the trend toward longer survival (median survival, 39 days vs 36 days; supplemental Table 5) suggests that this may be achievable by modifying the treatment regimen. Consistent with longer survival being a consequence of a reduction in tumor burden, flow cytometric analysis of the intrafemoral bone marrow taken on day 28 during treatment (Figure 6C) showed a reduction in transplanted MV4;11 (human CD45^+^) cells when compared with controls. The positive response for this aggressive, long-cultured AML CL suggests that an effective MNT inhibitor would be helpful for treating human *MLL-r* AMLs. Of note, many other AMLs in the BEAT AML data set have MNT levels at least as high as *MLL-r* AMLs (Figure 6D) and thus may also be dependent on MNT.

## Discussion

The last 2 decades have seen significant progress in identifying diverse genetic driver mutations in AML and in developing therapies directed against those mutations, such as inhibitors of FLT3 (FMS-related tyrosine kinase 3), IDH1/IDH2 (Isocitrate dehydogenase 1/2), BCL-2, and MENIN, and epigenetic modifiers.^2^^,^^47^^,^^49^, ^50^, ^51^, ^52^, ^53^, ^54^ Although targeted therapeutics may have only modest activity when used as single agents, they can significantly augment clinical efficacy when administered in combination with other drugs.^49^ Treatment with the BCL-2 inhibitor venetoclax, in combination with azacytidine or decitabine^47^^,^^51^^,^^55^ or with low dose cytarabine,^56^^,^^57^ is now approved for treatment of newly diagnosed patients with AML who are ineligible for intensive chemotherapy, and MCL-1 inhibitors have entered clinical trials.^58^ However, although venetoclax combination therapy has improved the response rates and reduced toxicities, most patients with AML still relapse within 8 to 17 months.^51^^,^^57^ Hence, despite these advances, there is still an urgent need for additional therapeutic agents to improve the outcomes for patients with AML.

Here, we have established for the first time to our knowledge, the dependency of certain myeloid leukemias on the bHLHLZ transcription factor MNT. Using multiple independent primary murine *MLL::AF9* AMLs, we showed in vitro that *Mnt* deletion provoked apoptosis and augmented the cell death triggered by the BH3 mimetics S63845 (MCL-1 inhibitor; *P* < .0001), ABT-199/venetoclax (BCL-2 inhibitor; *P* < .0001), and A-1331852 (BCL-X_L_ inhibitor; *P* < .01). In vivo, we found that *Mnt* deletion significantly extended the survival of mice that were transplanted with *MLL-AF9* AMLs (*P* < .0001; median survival, 57 days vs 17 days). Most notably, 11 of the 22 tamoxifen-treated *Mnt*^*fl/fl*^ *CreERT2/MLL::AF9* transplant recipients seemed to be cured, because their blood and bone marrow profiles had returned to normal by the time the experiment was terminated (195 days). The greater efficacy of *Mnt* deletion in vivo when compared with in vitro may be a consequence of the tumor environment in vivo.

To extend our studies to human AML, we used doxycycline-inducible CRISPR/Cas9^39^^,^^40^ to delete *MNT* from 4 long-established human AML CLs, namely MV4;11, which harbors an *MLL::AF4* fusion gene; MOLM-13 and THP-1, which have *MLL::AF9* fusion genes; and OCI-AML3, which has the *NPM1* and *DNMT3A* mutations that are common in adult AML.^59^ MNT reduction in MV4;11 and, to a lesser extent, in THP-1 and OCI-AML3 rendered these CLs more sensitive to killing by the MCL-1 inhibitor S63845 and, in the case of MV4;11, also by the BCL-2 inhibitor ABT-199/venetoclax. Importantly, we also showed that inducing *MNT* deletion in MV4;11 cells transplanted into immunodeficient NSG mice significantly enhanced the survival of transplant recipients.

In conclusion, this study has established, for the first time to our knowledge, that primary murine *MLL::AF9* AMLs and long-established human AML CLs are MNT dependent. These findings extend those we have made previously about the MNT dependency of MYC-driven B and T lymphomas, which we have shown was a consequence of MNT-mediated suppression of MYC-driven apoptosis, at least in part, via reduced expression of proapoptotic BIM.^25^^,^^26^

The deregulated overexpression of MYC is critical for driving many human cancers^60^, ^61^. However, to date, MYC has proven to be an intractable target for the development of clinically effective inhibitors.^62^ Our findings present an entirely new avenue to explore. Rather than inhibiting MYC, we propose taking advantage of MYC’s innate capacity to induce apoptosis and to amplify that drive by inhibiting MNT (or its regulators). To progress this possibility, it will be important to investigate which other tumor types are MNT dependent.

Possible approaches to MNT inhibition include inhibiting its heterodimerization to MAX; inhibiting MNT/MAX DNA binding; inhibiting MNT from binding to its vital co-repressor SIN3; and a PROTAC (Proteolysis Targeting Chimaera) strategy for targeting the degradation of MNT protein. MNT inhibitors are likely to be more effective in combination with additional drugs, and our results suggest that they could be a useful adjunct to BH3 mimetic drug therapy.

Conflict-of-interest disclosure: Walter and Eliza Hall Institute and its employees benefit from milestone and royalty payments related to venetoclax (ABT-199). G.L.K. and A.H.W. report receiving research funding from Servier for work on the development of S63845. The remaining authors declare no competing financial interests.

## Supplementary Material

Supplemental Figures, Tables, Methods, and References
