# Supplementary material for: MNT: a new target for AML
Source: Blood Neoplasia. 2025 Aug 4;2(4):100149. doi: 10.1016/j.bneo.2025.100149 (PMC12549552; doi:10.1016/j.bneo.2025.100149)
Supplement: Supplemental Figures, Tables, Methods, and References [file BNEO_NEO-2025-000579-mmc1.pdf]

Fig. S1 Fischer *et al.*

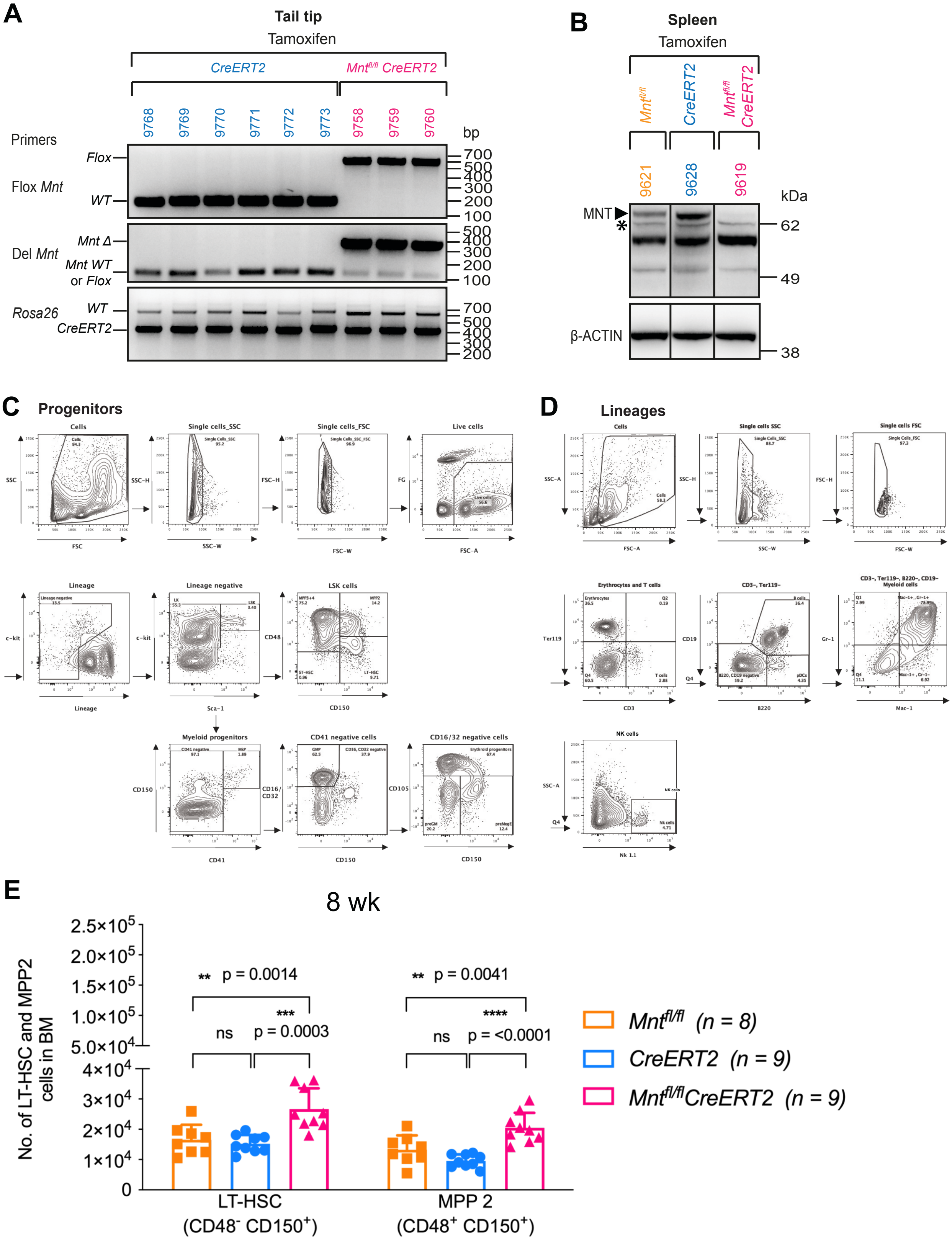

Figure S1. MNT loss has only modest impact on normal haemopoiesis. (A, B**)** Deletion of floxed *Mnt* alleles by CRE-ERT2 enzyme. (A) PCR of DNA from tail tip tissue showing efficient deletion of floxed *Mnt* alleles by *CreERT2* (presence of 386-bp *Mnt*Δ fragment in tail tip tissue of *Mnt*$^{fl/fl}$ *CreERT2* mice (9785, 9759, 9760) but not in tissues of *CreERT2* mice (9768-9773). (B**)** Western blot analysis of lysates of spleen cells obtained from individual tamoxifen-treated mice of the indicated genotypes. In (A), PCR primers and DNA markers (bp) are shown. In (B), arrow indicates MNT band, asterisk indicates non-specific band; MW markers (kD) are shown. (C, D) Gating strategies for bone marrow analysis. Results shown are from a female *Mnt*$^{fl/fl}$ *CreERT2* mouse 4 weeks post tamoxifen treatment. (C) Representative flow cytometric analysis of progenitor cell populations: Lin^{-} Sca-1^{+} c-Kit^{+} (LSK) cells, CD48^{-} CD150^{+} long-term (LT) HSCs, CD48^{-} CD150^{-} short-term (ST) HSCs, CD48^{+} CD150^{-} MPP 3+4 cells, CD48^{+} CD150^{-} MPP 2 cells, Lin^{-} Sca-1^{-} c-Kit^{+} (LK) cells, pre-granulocyte-macrophage progenitors (pre-GM), granulocyte macrophage progenitors (GMP), megakaryocyte progenitors (MKp), pre-megakaryocyte-erythroid progenitors (preMegE) and erythroid progenitors (Ep). (D) Representative flow cytometric analysis of mature cell lineages: B and T lymphoid cells, Mac-1^{+}Gr-1^{+} and Mac-1^{+}Gr-1^{-} myeloid cells, nucleated erythrocytes and natural killer (NK) cells. (E) Quantification of LT-HSC and MPP2 progenitor cells in bone marrow of *Mnt*$^{fl/fl}$ (orange), *CreERT2* (blue) and *Mnt*$^{fl/fl}$ *CreERT2* (pink) mice respectively, 8 weeks post tamoxifen treatment (see also Supplemental Table S1) Results are presented as mean ± SD from 8-9 mice of each genotype from 5 independent experiments. ns p>0.05, **p≤0.01, ***p≤0.001 and ****p≤0.0001 as determined by One-way ANOVA with Tukey's multiple comparison test.

Fig. S2 Fischer *et al.*

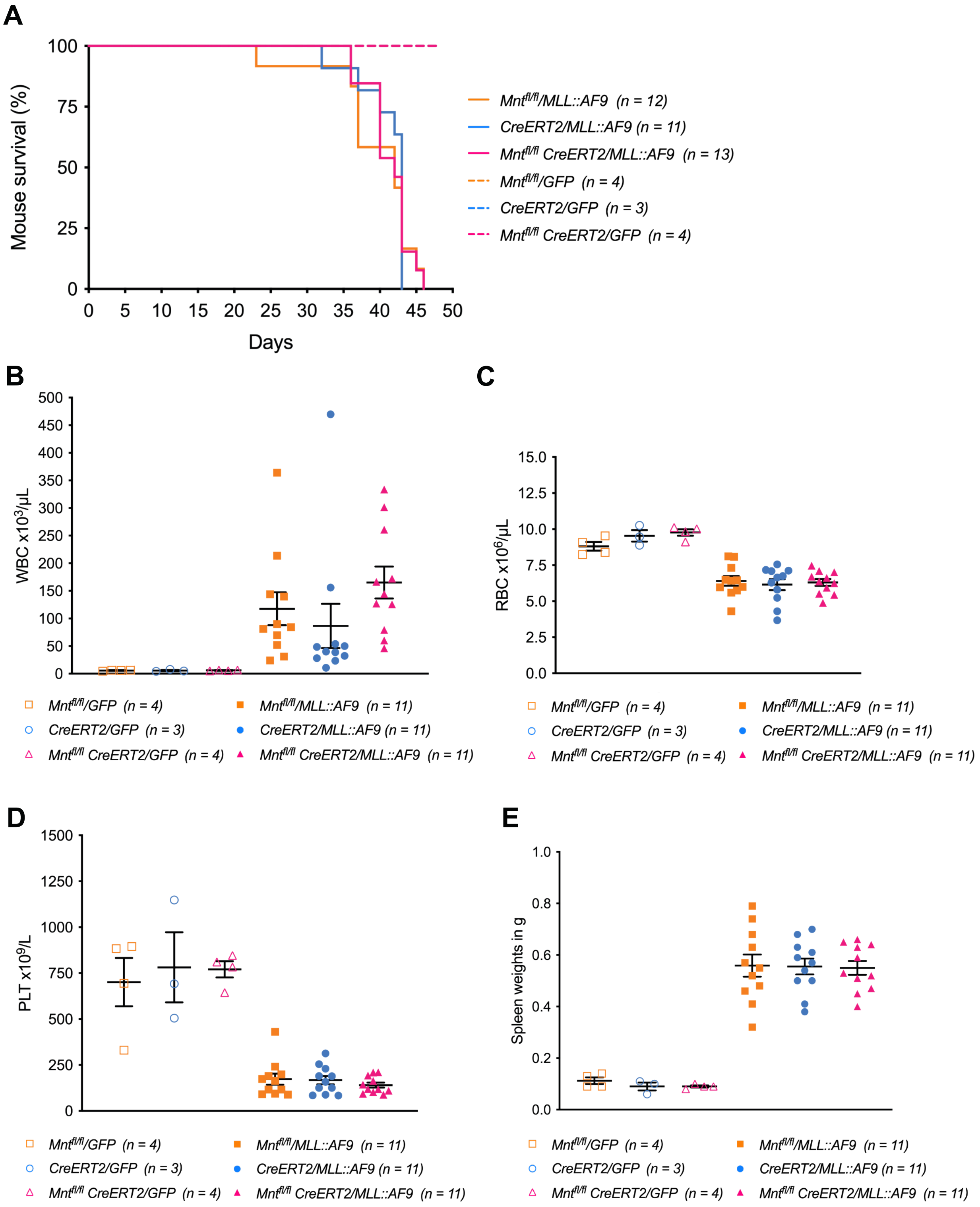

Figure S2. Generation of murine *MLL::AF9* AMLs for conditional *Mnt* deletion. (A) Kaplan–Meier plot showing the survival of mice reconstituted with foetal liver cells of the indicated genotypes infected with either *MLL::AF9* or *GFP* control viruses: $Mnt^{fl/fl}$/*MLL::AF9* (orange, *n* = 12), *CreERT2*/*MLL::AF9* (blue, *n* = 11), $Mnt^{fl/fl}$ *CreERT2*/*MLL::AF9* (pink, *n* = 13), $Mnt^{fl/fl}$/*GFP* (orange, dashed line n = 4), *CreERT2/GFP* (blue, dashed line n = 3) or $Mnt^{fl/fl}$ *CreERT2/GFP* (pink, dashed line n = 4) . Mice were monitored regularly and euthanised when their symptoms dictated ethical endpoint. Control GFP mice remained healthy until humanely euthanised 7 weeks post-transplantion. (B-E) WBC, RBC and platelet counts, and spleen weights of individual *MLL::AF9* AML and GFP control mice of the indicated genotypes at autopsy. Data points represent individual mice with mean ± SEM indicated.

Fig. S3 Fischer *et al.*

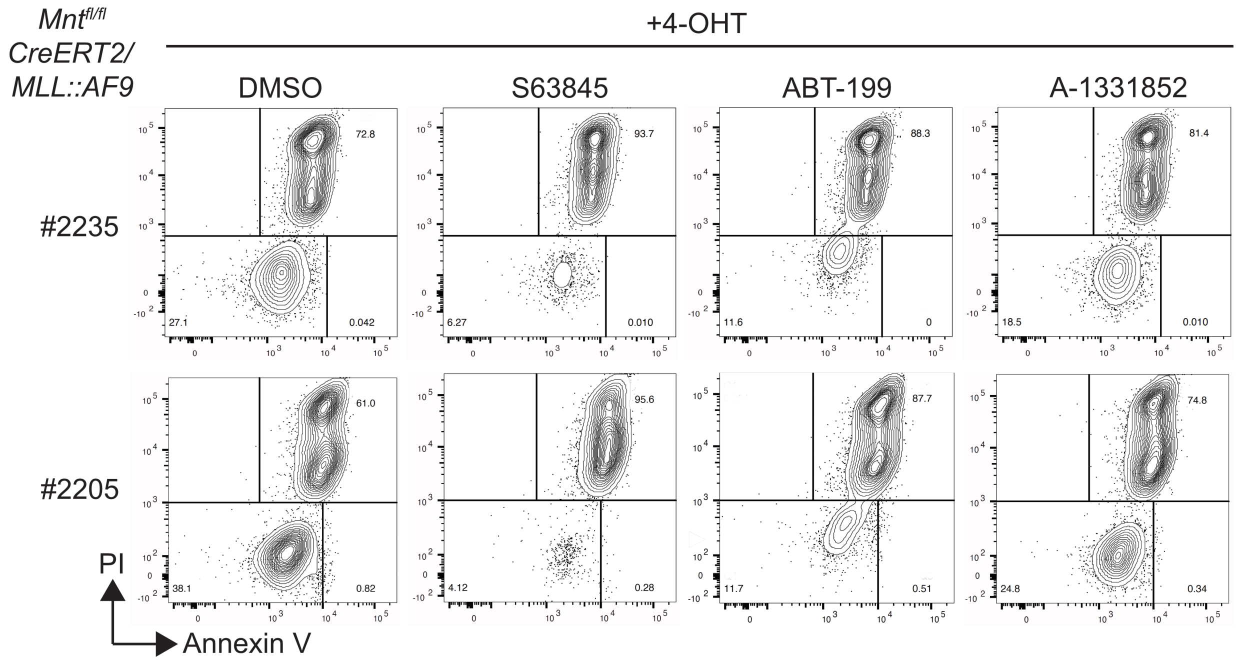

Figure S3. ***Mnt*** **deletion enhances sensitivity of** ***MLL::AF9*** **AML cell lines to BH3 mimetic drugs S63845, ABT-199 and A-1331852.** Flow cytometric viability analysis of two representative *Mnt*$^{fl/fl}$ *CreERT2/MLL::AF9* cell lines (#2235 and #2205) treated with 4-OHT and BH3 mimetic drugs S63845 (MCL-1 inhibitor), ABT-199/venetoclax (BCL-2 inhibitor) or A-1331852 (BCL-X_L inhibitor). Percentages of live (Annexin V^-/PI^-), early apoptotic (Annexin V^+/PI^-) and late apoptotic (Annexin V^+/PI^+) cell populations are indicated.

Table S1: *Mnt* deletion *in vivo* results in modest changes in haemopoietic tissues.

	4 weeks				8 weeks			
**Peripheral blood**	***Mnt*$^{fl/fl}$ (n = 11)**	***Mnt*$^{+/+}$ *CreERT2* (n = 10)**	***Mnt*$^{fl/fl}$ *CreERT2* (n = 10)**	**P value**	***Mnt*$^{fl/fl}$ (n = 8)**	***Mnt*$^{+/+}$ *CreERT2* (n = 9)**	***Mnt*$^{fl/fl}$ *CreERT2* (n = 8)**	**P value**
WBC $x10^3$/µL	7.47 ± 1.60	7.45 ± 1.21	5.02 ± 0.86	****	5.71 ± 1.28	6.46 ± 0.71	5.48 ± 1.43	ns
RBC $x10^6$/µL	9.88 ± 0.57	10.11 ± 0.34	9.18 ± 0.70	**	9.78 ± 0.45	10.30 ± 0.17	9.93 ± 0.19	***
Haemoglobin g/dL	15.65 ± 0.80	15.88 ± 0.50	14.91 ± 1.07	*	15.6 ± 0.60	15.9 ± 0.22	15.8 ± 0.32	ns
Haematocrit %	51.37 ± 2.89	51.6 ± 1.71	49.53 ± 2.51	*	51.3 ± 2.5	51.3 ± 1.0	52.3 ± 0.9	ns
Platelets $x10^6$/µL	1088 ± 136	1229 ± 227	1086 ± 163	ns	1150 ± 150	1210 ± 160	1270 ± 160	ns
Lymphocytes $x10^3$ cells/µL	6.31 ± 1.54	6.29 ± 1.03	3.93 ± 0.51	****	4.66 ± 1.16	5.58 ± 0.55	4.53 ± 1.14	*
Neutrophils $x10^3$ cells/µL	0.83 ± 0.22	0.81 ± 0.22	0.85 ± 0.56	ns	0.75 ± 0.27	0.61 ± 0.18	0.67 ± 0.22	ns
Eosinophils $x10^3$ cells/µL	0.11 ± 0.04	0.13 ± 0.04	0.11 ± 0.04	ns	0.13 ± 0.04	0.09 ± 0.03	0.13 ± 0.03	*
Monocytes $x10^3$ cells/µL	0.11 ± 0.05	0.13 ± 0.04	0.07 ± 0.03	**	0.09 ± 0.02	0.09 ± 0.03	0.08 ± 0.03	ns
Basophils $x10^3$ cells/µL	0.04 ± 0.03	0.03 ± 0.02	0.02 ± 0.01	ns	0.02 ± 0.01	0.05 ± 0.04	0.03 ± 0.02	ns
Large peroxidase-negative cells (LUC) $x10^3$ cells/µL	0.05 ± 0.02	0.05 ± 0.02	0.03 ± 0.02	*	0.04 ± 0.01	0.05 ± 0.02	0.05 ± 0.02	ns
**Bone marrow**	***Mnt*$^{fl/fl}$ (n = 11)**	***Mnt*$^{+/+}$ *CreERT2* (n = 10)**	***Mnt*$^{fl/fl}$ *CreERT2* (n = 10)**	**P value**	***Mnt*$^{fl/fl}$ (n = 5-8)**	***Mnt*$^{+/+}$ *CreERT2* (n = 9)**	***Mnt*$^{fl/fl}$ *CreERT2* (n = 7-9)**	**P value**
Total cellularity	93.73 ± 20.01	104.70 ± 20.03	89.80 ± 16.02	ns	112.00 ± 15.59	106.00 ± 20.81	131.30 ± 30.86	ns
B cells (Ter119$^-$ CD3$^-$ CD19$^+$ B220$^+$)	9.88 ± 3.11	11.24 ± 3.10	8.39 ± 2.37	*	9.94 ± 2.25	8.57 ± 1.50	9.88 ± 1.83	ns
T cells (Ter119$^-$ CD3$^+$)	1.09 ± 0.27	1.22 ± 0.29	1.00 ± 0.21	ns	1.64 ± 0.62	1.26 ± 3.42	1.59 ± 3.26	ns
Nucleated erythrocytes (Ter-119$^+$)	15.78 ± 3.05	16.26 ± 3.77	16.14 ± 3.15	ns	18.3 ± 3.6	15.5 ± 3.1	21.7 ± 3.9	**
NK cells (Ter119$^-$ CD3$^-$ CD19$^-$ B220$^-$ Mac-1$^-$ Gr-1$^-$ Nk1.1$^+$)	0.08 ± 0.02	0.1 ± 0.02	0.05 ± 0.01	****	0.11 ± 0.04	0.12 ± 0.03	0.10 ± 0.04	ns
Plasmacytoid dendritic cells (pDCs) (Ter119$^-$ CD3$^-$ CD19$^-$ B220$^+$)	1.18 ± 0.48	1.01 ± 0.25	1.00 ± 0.78	ns	1.49 ± 0.49	0.90 ± 0.22	1.10 ± 3.73	ns

Mac-1$^+$ Gr-1$^+$ myeloid cells (Ter119$^-$ CD3$^-$ CD19$^-$ B220$^-$)	13.86 ± 4.14	17.26 ± 4.86	13.59 ± 4.8	ns	14.7 ± 3.38	18.3 ± 5.12	22.6 ± 9.42	ns
Mac-1$^+$ Gr-1$^-$ myeloid cells (Ter119$^-$ CD3$^-$ CD19$^-$ B220$^-$)	0.83 ± 0.12	1.88 ± 0.35	1.07 ± 0.20	ns	1.30 ± 0.12	1.36 ± 0.23	1.81 ± 0.45	*
Lin$^-$ Sca-1$^+$ cKit$^+$ (LSK) cells	195064 ± 65849	180243 ± 40051	181548 ± 44854	ns	182000 ± 33700	134000 ± 035000	212000 ± 39800	***
Lin$^-$ Sca-1$^-$ cKit$^+$ (LK) cells	3271244 ± 555729	4153386 ± 1004032	2936966 ± 720552	**	4.30 ± 0.936	3.27 ± 1.10	4.38 ± 1.27	ns
Lin$^-$ Sca-1$^+$ cKit$^+$ CD48$^+$ CD150$^-$ (MPP 3+4) cells	152427 ± 51358	142238 ± 33623	138704 ± 43921	ns	148518 ± 29869	106953 ± 31772	161496 ± 35958	**
Lin$^-$ Sca-1$^+$ cKit$^+$ CD48$^+$ CD150$^+$ (MPP 2) cells	18847 ± 11322	14052 ± 2655	22557 ± 7156	**	13275 ± 4722	9568 ± 2172	20493 ± 4959	****
Lin$^-$ Sca-1$^+$ cKit$^+$ CD48$^-$ CD150$^+$ (LT-HSC) cells	19859 ± 5629	20743 ± 4982	17752 ± 3948	ns	16536 ± 4965	15236 ± 2919	26710 ± 6821	***
Lin$^-$ Sca-1$^+$ cKit$^+$ CD48$^-$ CD150$^-$ (ST-HSC) cells	3885 ± 1307	3116 ± 1491	2575 ± 2778	ns	4243 ± 1179	2102 ± 1254	2904 ± 1810	ns
Pre-granulocyte-macrophage progenitors (preGM) (Lin$^-$ cKit$^+$ CD41$^-$ CD16/32$^-$ CD105$^-$ CD150$^-$)	277335 ± 65546	375680 ± 132158	274856 ± 133885	ns	291000 ± 81300	336000 ± 24000	42300 ± 26600	ns
Granulocyte-Macrophage progenitors (GMP) (Lin$^-$ cKit$^+$ CD41$^-$ CD16/32$^+$)	2163197 ± 446878	3056904 ± 892122	1877149 ± 517202	**	3.02 ± 0.77	2.29 ± 0.82	2.90 ± 1.01	ns
Megakaryocyte progenitors (MKp) (Lin$^-$ cKit$^+$ CD41$^+$ CD150$^+$)	63909 ± 12960	74548 ± 14062	52602 ± 12418	**	70700 ± 14400	42900 ± 16200	73000 ± 11400	***
Pre-megakaryocyte-erythroid progenitor (preMegE) (Lin$^-$ cKit$^+$ CD41$^-$ CD16/32$^-$ CD105$^-$ CD150$^+$)	143954 ± 46543	156645 ± 28673	150015 ± 38542	ns	159000 ± 27100	125000 ± 25900	207000 ± 35100	****
Erythrocyte progenitors (Ep) (Lin$^-$ cKit$^+$ CD41$^-$ CD16/32$^-$ CD105$^+$)	601160 ± 95217	474341 ± 97925	572915 ± 123228	ns	698000 ± 96600	429000 ± 121000	711000 ± 222000	**
**Spleen**	***Mnt$^{fl/fl}$* (n = 8)**	***Mnt$^{+/+}$ CreERT2* (n = 8)**	***Mnt$^{fl/fl}$ CreERT2* (n = 7)**	**P value**	***Mnt$^{fl/fl}$* (n = 5)**	***Mnt$^{+/+}$ CreERT2* (n = 8)**	***Mnt$^{fl/fl}$ CreERT2* (n = 7)**	**P value**
Total cellularity	167.50 ± 88.60	155.00 ± 46.44	132.60 ± 66.65	ns	152.40 ± 40.47	113.30 ± 25.03	110.00 ± 50.04	ns

B cells (Ter119$^-$ CD3$^-$ CD19$^+$ B220$^+$)	37.16 ± 21.79	31.75 ± 8.56	23.40 ± 13.61	ns	29.5 ± 7.1	24.7 ± 8.3	18.7 ± 7.2	ns
T cells (Ter119$^-$ CD3$^+$)	16.05 ± 9.63	11.86 ± 3.73	10.33 ± 5.17	ns	12.9 ± 3.6	10.4 ± 4.6	7.55 ± 4.0	ns
Nucleated erythrocytes (Ter-119$^+$)	9.20 ± 10.97	3.00 ± 1.54	6.42 ± 7.12	ns	2.6 ± 1.2	1.87 ± 0.94	0.95 ± 0.53	*
NK cells (Ter119$^-$ CD3$^-$ CD19$^-$ B220$^-$ Mac-1$^-$ Gr-1$^-$ Nk1.1$^+$)	0.18 ± 0.06	0.14 ± 0.05	0.08 ± 0.06	ns	0.21 ± 0.11	0.13 ± 0.09	0.05 ± 0.03	ns
Plasmacytoid dendritic cells (pDCs) (Ter119$^-$ CD3$^-$ CD19$^-$ B220$^+$)	1.78 ± 1.71	0.05 ± 0.02	1.24 ± 1.74	ns	0.66 ± 0.25	0.29 ± 0.10	0.31 ± 0.28	ns
Mac-1$^+$ Gr-1$^+$ myeloid cells (Ter119$^-$ CD3$^-$ CD19$^-$ B220$^-$)	1.61 ± 1.77	0.69 ± 0.24	1.59 ± 1.64	ns	1.31 ± 0.60	0.44 ± 0.13	0.57 ± 0.42	ns
Mac-1$^+$Gr-1$^-$ myeloid cells (Ter119$^-$ CD3$^-$ CD19$^-$ B220$^-$)	0.82 ± 0.27	0.84 ± 0.22	0.61 ± 0.29	ns	0.92 ± 0.27	0.78 ± 0.26	0.50 ± 0.19	*

Haemopoietic analysis was performed 4 and 8 wk post tamoxifen treatment using Advia 2120 for peripheral blood and flow cytometry for bone marrow and spleen (see Supplemental Fig. S2 for cell surface staining and sorting strategy). Values given are total nucleated cells x10^6 except for peripheral blood (as indicated) and LSK, MPP3+4, MPP2, LT-HSCs, ST-HSCs, preGM, MKp, preMegE and Ep, which are total numbers.

Data are expressed as mean ± SD for 7-11 mice (4 wk timepoint) and 5-9 mice (8 ws timepoint) of each genotype as indicated, analysed in 5 independent experiments; ns $p>0.05$, *$p\leq 0.05$, **$p\leq 0.01$, ***$p\leq 0.001$ and ****$p\leq 0.0001$ as determined by unpaired t-test comparing Mnt$^{fl/fl}$ CreERT2 to Mnt$^{+/+}$CreERT2.

Table S2. Generation of primary *MLL::AF9* AMLs[1]

Ly5.2 E14 embryo genotype, sex	Virus	Recipient mouse # sex	Survival (d)[2]	WBC at autopsy[3] $x10^3$ cells/µl	RBC at autopsy[3] $x10^6$ cells/µl	PLT at autopsy[3] $x10^9$ cells/L	Spleen (mg) at autopsy	Liver (gm) at autopsy
$Mnt^{fl/fl}$ F	*MLL::AF9*	#2231 F	40	301	7.5	193	630	1.75
$Mnt^{fl/fl}$ F	*MLL::AF9*	#2230 F	40	nd	nd	nd	440	1.22
$Mnt^{+/+}$ *CreERT2* M	*MLL::AF9*	#2244 M	40	470	4.3	230	590	1.67
$Mnt^{+/+}$ *CreERT2* M	*MLL::AF9*	#2243 M	42	54	5.8	85	700	2.21
$Mnt^{+/+}$ *CreERT2* F	*MLL::AF9*	#2201 F	37	50	6.8	255	500	1.75
$Mnt^{+/+}$ *CreERT2* M	*MLL::AF9*	#2242 M	42	39	7.2	158	680	1.90
$Mnt^{+/+}$ *CreERT2* M	*MLL::AF9*	#2210 M	32	48	6.7	125	410	1.29
				**Median 50**	**Median 6.7**	**Median 158**	**Median 590**	**Median 1.75**
$Mnt^{fl/fl}$ *CreERT2* F	*MLL::AF9*	#2232 F	44	166	5.9	102	650	1.57
$Mnt^{fl/fl}$ *CreERT2* F	*MLL::AF9*	#2205 F	36	333	6.3	163	510	1.90
$Mnt^{fl/f}$ *CreERT2* M	*MLL::AF9*	#2214 M	43	144	7.0	110	400	1.31
$Mnt^{fl/fl}$ *CreERT2* F	*MLL::AF9*	#2206 F	43	46	6.6	88	450	1.48
$Mnt^{fl/fl}$ *CreERT2* F	*MLL::AF9*	#2235 F	42	126	5.4	118	470	1.58
$Mnt^{fl/fl}$ *CreERT2* F	*MLL::AF9*	#2233 F	41	127	4.9	209	660	1.71
				**Median 136**	**Median 6.1**	**Median 114**	**Median 490**	**Median 1.58**
$Mnt^{fl/fl}$ M	*GFP*	#2219 M	48	6.5	8.4	883	90	1.09
$Mnt^{fl/fl}$ M	*GFP*	#2220 M	48	6.0	9.1	695	130	1.24
$Mnt^{fl/fl}$ F	*GFP*	#2238 F	47	6.3	9.5	895	90	1.15
$Mnt^{fl/fl}$ F	*GFP*	#2239 F	47	4.6	8.2	331	140	0.97
				**Median 6.15**	**Median 8.8**	**Median 789**	**Median 110**	**Median 1.12**
$Mnt^{+/+}$*CreERT2* M	*GFP*	#2217 M	48	4.9	9.5	1148	60	1.31
$Mnt^{+/+}$*CreERT2* M	*GFP*	#2218 M	48	7.7	10	692	110	1.08
				**Median 6.3**	**Median 9.8**	**Median 920**	**Median 85**	**Median 1.20**
$Mnt^{fl/fl}$ *CreERT2* M	*GFP*	#2221 M	48	5.6	9.8	811	90	1.85
$Mnt^{fl/fl}$ *CreERT2* M	*GFP*	#2222 M	48	5.1	9.1	846	100	1.52
$Mnt^{fl/fl}$ *CreERT2* F	*GFP*	#2240 F	47	6.6	10	644	80	0.91
$Mnt^{fl/fl}$ *CreERT2*	*GFP*	#2241 F	47	6.7	10	783	90	1.22
				**Median 5.9**	**Median 9.9**	**Median 797**	**Median 90**	**Median 1.37**

[1]13 independent primary murine AMLs were generated by infection of Ly5.2 C57BL/6 foetal liver cells with *MLL::AF9* retrovirus, followed by transplantation into sub-lethally irradiated syngeneic Ly5.1 mice. Mice were humanely euthanised when reaching ethical endpoint. 10 sub-lethally irradiated control mice were transplanted with foetal liver cells infected with GFP retrovirus followed by transplantation.

[2] Survival of mice developing AML ranged from 32 to 42 d. All control mice remained healthy until humanely euthanised 47 or 48 d post-transplant.

[3]Blood was taken by retro-orbital bleed at point of euthanasia and analysed using Advia 2120. Normal range for WBC, RBC and platelets (PLT) is WBC 7.7±1.2 $x10^3$ cells/µl, RBC 10.11±0.34 $x10^6$ cells/µl and PLT 1229±226 $x10^9$ cells/L respectively.

Table S3. Impact of *Mnt* loss on survival of mice transplanted with primary *MLL::AF9* AMLs

Genotype	1^0 AML	Transplant recipients	Treatment	2 wk WBC $x10^3$ cells/µl	2 wk PLT $x10^9$ cells/L	Survival (d)	Autopsy WBC $x10^3$ cells/µl	Autopsy PLT $x10^3$ cells/L	Autopsy BM: *Mnt* genotype	Autopsy BM: MNT protein	Autopsy BM: MYC protein
***Mnt*$^{fl/fl}$**	**#2231**<br>(F)<br>Exp 2	#9845-48<br>(F)<br>#9849-52<br>(F)	vehicle<br><br>tamoxifen	19.2,*,20.5,16.1<br>**median 17.6**<br>23.2,25.1,30.6,34.1<br>**median 27.8**	440,*,590,420<br>**median 450**<br>420,310,300,330<br>**median 305**	17, *,17,17<br>**median 17**<br>16,15,16,17<br>**median 16**	75,*,125,77<br>**median 75**<br>208,102,102,122<br>**median 102**	172,*,282,210<br>**median 210**<br>562,271,248,211<br>**median 260**	*fl/fl, fl/fl, fl/fl,fl/fl*<br><br>*fl/fl, fl/fl, fl/fl,fl/fl*	+,+,+,+<br><br>+,+,+,+	hi, hi,hi,hi<br><br>hi,hi,hi,hi
	**#2230**<br>(F)<br>Exp 5	#9971-74<br>(F)<br>#9975-78<br>(F)	vehicle<br><br>tamoxifen	8.1,*,10.5, 8.3<br>**median 8.3**<br>7.0,9.4,5.3<br>**median 7.0**	1010,*,840,980<br>**median 980**<br>930,940,620<br>**median 930**	24,*,20,20<br>**median 20**<br>*,21,41,23<br>**median 23**	192, *,204,104<br>**median 104**<br>179,300,209<br>**median 209**	328, *,252,377<br>**median 328**<br>386,374,294<br>**median 374**	*fl/fl, fl/fl, fl/fl,fl/fl*<br><br>*fl/fl, fl/fl, fl/fl,fl/fl*	not done<br><br>not done	not done<br><br>not done
CreERT2	**#2244**<br>(M)<br>Exp 2	#9827-30<br>(M)<br>#9831-34<br>(M)	vehicle<br><br>tamoxifen	21.2,24.6,24.8,35.4<br>**median 24.7**<br>5.1,5.4,9.1,4.4<br>**median 5.3**	960,970,620,700<br>**median 830**<br>590,640,790,810<br>**median 715**	19,19,19,19<br>**median 19**<br>24,24,24,24<br>**median 24**	328,385,425,345<br>**median 365**<br>283,405,414,508<br>**median 410**	198,221,183,182<br>**median 190**<br>251,308,222,270<br>**median 237**	+/+, +/+, +/+,+/+<br><br>+/+, +/+, +/+,+/+	+,+,+,+<br><br>+,+,+,+	hi, hi,hi,hi<br><br>hi,hi,hi,hi
	**#2243**<br>(M)<br>Exp3	#9865-68<br>(M)<br>#9869-72<br>(M)	vehicle<br><br>tamoxifen	19.1,22.5,20.2,35.9<br>**median 21.3**<br>7.5,6.9,6.1,4.8<br>**median 6.5**	920,1000,740,340<br>**median 830**<br>1050,1080,950,1090<br>**median 1065**	17,17,17,17<br>**median 17**<br>22,22,25,25<br>**median 23.5**	103,113,120,189<br>**median 117**<br>69,137,78,94<br>**median 86**	11,12,11,128<br>**median 11.5**<br>224,230,174,148<br>**median 199**	+/+, +/+, +/+,+/+<br><br>+/+, +/+, +/+,+/+	+,+,+,+<br><br>+,+,+,+	hi, hi,hi,hi<br><br>hi,hi,hi,hi
	**#2201**<br>(F)<br>Exp 4	#9899-9902<br>(F)<br>#9903-06<br>(F)	vehicle<br><br>tamoxifen	7.4,5.1,9.3,13.1<br>**median 8.3**<br>6.1,6.8,8.2,6.9<br>**median 6.8**	1200,1170,1190,1160<br>**median 1180**<br>830,860,920,760<br>**median 845**	23,23,23,20<br>**median 23**<br>28,26,28,28<br>**median 28**	103,135,117,116<br>**median 117**<br>79,81,112,92<br>**median 87**	283,327,298,393<br>**median 313**<br>256,341,244,231<br>**median 250**	+/+, +/+, +/+,+/+<br><br>+/+, +/+, +/+,+/+	+,+,+,+<br><br>+,+,+,+	hi, hi,hi,hi<br><br>hi,hi,hi,hi
	**#2242**<br>(M)<br>Exp 5	#9955-58<br>(M)<br>#9959;#9961-63<br>(M)	vehicle<br><br>tamoxifen	16.4,18.7,26.3,22.2<br>**median 20.4**<br>4.3,6.1,7.5,6.6<br>**median 6.3**	1010,1210,730,1080<br>**median 1045**<br>820,760,1000,1140<br>**median 910**	18,17,17,18<br>**median 17.5**<br>25,24,24,24<br>**median 24**	202,149,160,155<br>**median 152**<br>229,215,268,219<br>**median 224**	252,389,359,280<br>**median 320**<br>229,323,313,365<br>**median 318**	+/+, +/+, +/+,+/+<br><br>+/+, +/+, +/+,+/+	+, +, +, +<br><br>+, +, +, +	hi,hi,hi,hi<br><br>hi,hi,hi,hi
	**#2210**<br>(M)<br>Exp 6	#9990-93<br>(M)<br>#9994-97<br>(M)	vehicle<br><br>tamoxifen	42.8,33.5,39.7,34.1<br>**median 36.9**<br>6.7,7.6,7.0,8.5<br>**median 7.3**	682,738,840,840<br>**median 789**<br>960,1118,1052,838<br>**median 1006**	17,15,17,17<br>**median 17**<br>21,21,23,23<br>**median 22**	113,33,154,164<br>**median 134**<br>102,131,131,164<br>**median 131**	303,691,345,321<br>**median 333**<br>170,181,175,316<br>**median 178**	+/+, +/+, +/+,+/+<br><br>+/+, +/+, +/+,+/+	+, +, +, +<br><br>+, +, +, +	hi, hi,hi,hi<br><br>hi,hi,hi,hi
Mnt*$^{fl/fl}$**<br>CreERT2***	**#2232**<br>(F)<br>Exp 1	#9807-9810<br>(F)<br>#9811-14<br>(F)	vehicle<br><br>tamoxifen	13.3,18.3,21.7,22.5<br>**median 20**<br>5.9,7.2,8.4,5.9<br>**median 6.5**	860,480,320,450<br>**median 465**<br>900,630,660,890<br>**median 775**	17,17,17,17<br>**median 17**<br>38,29,26,28<br>**median 28.5**	77,124,144,127<br>**median 126**<br>133,211,164,95<br>**median 148**	249,236,176,169<br>**median 206**<br>201,662,238,265<br>**median 252**	*fl/fl,fl/fl,fl/fl,fl/fl*<br><br>lost,-/-,-/-,-/-	+.+.+.+<br><br>-, lo,lo,lo	hi,hi,hi,hi<br><br>med,med,<br>med,med
	**#2205**<br>(F)<br>Exp 2	#9837-40<br>(F)<br>#9841-44<br>(F)	vehicle<br><br>tamoxifen	54.4,46,77,59.2<br>**median 56.8**<br>11.8,10.0,9.8,10.6<br>**median 10.3**	300,92,200,150<br>**median 175**<br>740,740,650,700<br>**median 720**	17,17,17,17<br>**median 17**<br>195,59,24,195<br>**median 127**	236,208,217,301<br>**median 227**<br>5.3, 55, 154,5.7<br>**median 30**	166,148,265,227<br>**median 197**<br>1224,293,188,1183<br>**median 738**	*fl/fl,fl/fl,fl/fl,fl/fl*<br><br>+/+,-/-,-/-,+/+	+,+,+,+<br><br>-, -, -, -	hi, hi,hi,hi<br><br>nd,hi,hi,lo
	**#2214**<br>(M)<br>Exp 3	#9873-76<br>(M)<br>#9877-80<br>(M)	vehicle<br><br>tamoxifen	26.2,18.2,16.3,23.9<br>**median 21.5**<br>5.8,8.0,7.3,8.4<br>**median 7.7**	730,570,800,760<br>**median 745**<br>1150,860,880,1110<br>**median 995**	18,19,17,20<br>**median 18.5**<br>30,30,30,55<br>**median 30**	82,114,72,131<br>**median 98**<br>149,160,350,153<br>**median 157**	374,397,262,344<br>**median 359**<br>354,424,322,358<br>**median 356**	*fl/fl,fl/fl,fl/fl,fl/fl*<br><br>-/-,-/-,-/-,-/-	+,+.+.+<br><br>-,-,-,-	hi, hi,hi,hi<br><br>hi,hi,hi,hi
	**#2206**<br>(F)<br>Expt 4	#9907-9910<br>(F)<br>#9911-9914<br>(F)	vehicle<br><br>tamoxifen	34.2,22.1,49.7,*<br>**median 34.2**<br>*,6.8,10.2,12.6<br>**median 10.2**	630,410,600,*<br>**median 600**<br>*,760,770,860<br>**median 770**	17,17,17, *<br>**median 17**<br>*,195,195,28<br>**median 85**	286,286,331,*<br>**median 286**<br>*, 5.3,5.1,236<br>**median 5.3**	269,197,234,*<br>**median 234**<br>*,1046,889,198<br>**median 889**	*fl/fl, fl/fl, fl/fl* *<br><br>*, +/+,+/+,-/-	+,+,+<br><br>*,-, -, -	hi,hi,hi,*<br><br>*,nd,nd,med

	**#2235**<br>(F)<br>Expt 5	#9979-82<br>(F)<br>#9983-86<br>(F)	vehicle<br><br>tamoxifen	8.2,9.0,7.0, †<br>**median 8.2**<br>8.1,8.7,9.6,7.8<br>**median 8.4**	1190,1070,1100, †<br>**median 1100**<br>760,730,970,880<br>**median 820**	20,20,20, †<br>**median 20**<br>195,195,195,195<br>**median >195**	211,155,148, †<br>**median 155**<br>7.9,7.0,7.7,8.6<br>**median 7.4**	270,244,218, †<br>**median 244**<br>937,875,1106,1290<br>**median 1022**	*fl/fl, fl/fl,fl/fl,†*<br><br>+/+, +/+, +/+,+/+	+, +, +, †<br><br>lo, lo, lo, lo	hi,med,hi, †<br><br>lo,lo.lo.lo
	**#2233**<br>(F)<br>Expt 6	#10014-17<br>(F)<br>#10018-21<br>(F)	vehicle<br><br>tamoxifen	8.8,25.6, †,64.1<br>**median 25.6**<br>7.4, †,,5.9,5.5<br>**median 5.5**	434,818, †,536<br>**median 536**<br>858, †,,852,944<br>**median 892**	25,17, †,17<br>**median 17**<br>195, †,195,195<br>**median 113**	110,134, †, fd<br>**median 122**<br>6.7, †,,6.6,7.2<br>**median 6.7**	251,400, †, fd<br>**median 326**<br>1197, †,,1109,1244<br>**median 1153**	*fl/fl,fl/fl,* †, fd<br><br>+/+, †, +/+, +/+	+, +, †, fd<br><br>lo, †, lo, lo	hi,hi, †,fd<br><br>lo, †,,lo,lo

Transplanted mice were treated with vehicle or tamoxifen as described in text and their health regularly monitored for up to 195d, when the experiment was terminated. Sick mice were euthanised and autopsied on days indicated. Mice remaining healthy on d195 (#9841, #9844, #9912, #9913, #9983-9986, #10018, #10020, #10021, marked in red) appeared to be in total remission as they had normal blood profiles (median WBC 6.5; median platelets 1069), low MYC protein and lacked detectable *CreERT2* or *Mnt*Δ band by PCR; for examples see #9841, #9844 in Fig. 3B,C).

* # 9846, #9972, #9910, #9911, #9975 were culled on d 17, d73, d59, d75, d24, and censored from survival curve because their morbidity was not due to AML but instead to issues such as infection.

† #9982, #10016, #1019 were censored from survival curve due to apparent injection error.

[1] #9875 had dominant wt *Mnt* band, suggesting lower AML content than others in #2214 (-tamoxifen) cohort

nd = not detectable

Table S4. Impact of combining *Mnt* deletion with BH3 mimetic treatment on survival of mice transplanted with 2º (T1) *MLL::AF9* AMLs

Genotype	†T0 /T1 AML	Recipients of T1 AML	§Tamoxifen	§S63845	2wk %GFP	2wk %Ly5.2	2 wk WBC $x10^3/\mu L$	2wk RBC $x10^6/\mu L$	2wk PLT $x10^9/L$	Survival (d)	Autopsy WBC $x10^3/\mu L$	Autopsy PLT $x10^9/L$
CreERT2	**#2201/#3378**	#10030	vehicle	vehicle	11	14	15	11	910	17	169	366
	(F)	#10031	vehicle	vehicle	7	6.6	15	11	880	17	118	361
	Expt1	#10032	vehicle	vehicle	14	22	22	11	530	17	125	187
		#10033	vehicle	vehicle	12	18	16	11	710	17	171	301
		#10034	vehicle	vehicle	9.9	17	28	12	780	17	158	323
		#10035	vehicle	vehicle	6.1	9.2	12	3	260	17	133	378
										**Mean 17**		
		#10036	vehicle	S63845	13	19	14	10	1490	17	147	390
		#10037	vehicle	S63845	16	24	20	9.5	1490	16	150	630
		#10038	vehicle	S63845	14	19	13	10	1580	17	137	468
		#10039	vehicle	S63845	29	39	22	8.7	1450	17	198	456
		#10040	vehicle	S63845	17	24	13	10	1670	17	157	526
		#10041	vehicle	S63845	13	15	11	10	1680	17	135	502
										**Mean 17**		
		#10042	tamoxifen	vehicle	1.9	0.2	10	11	870	23	150	276
		#10043	tamoxifen	vehicle	0.7	0.3	8.1	11	870	23	204	242
		#10044	tamoxifen	vehicle	0.6	0.2	7.0	11	950	24	151	224
		#10045	tamoxifen	vehicle	1.1	0.2	8.1	11	770	23	213	212
		#10046	tamoxifen	vehicle	0.8	0.2	7.6	10	840	24	145	196
		#10047	tamoxifen	vehicle	0.5	0.2	11	10	820	24	98	110
										**Mean 23.5**		
		#10048	tamoxifen	S63845	3.4	0.2	3.7	9.7	930	23	184	384
		#10049	tamoxifen	S63845	1.3	1.5	3.7	9.8	1530	23	186	330
		#10050	tamoxifen	S63845	4.1	0.2	5.8	10	1840	23	157	322
		#10051	tamoxifen	S63845	2.8	0.4	4.5	9.7	1620	23	212	386
		#10052	tamoxifen	S63845	1.5	0.3	3.8	10	1550	23	204	362
		#10053	tamoxifen	S63845	1.4	0.4	5.3	9.8	1370	23	185	320
										**Mean 23**		
Mnt$^{fl/fl}$* *CreERT2	**#2205/#3413**	#10054	vehicle	vehicle	43	66	97	9.6	200	15	225	215
	(F)	#10055	vehicle	vehicle	21	23	11	11	310	15	fd	fd
	Expt 1	#10056	vehicle	vehicle	33	47	66	11	230	15	fd	fd
		#10057	vehicle	vehicle	29	46	45	11	300	15	100	178
		#10058	vehicle	vehicle	31	45	69	7.5	460	15	183	210
		#10059	vehicle	vehicle	3.2	2.0	5.1	11	360	17	6.1	443
										**Mean 15**		
		#10060	vehicle	S63845	4.2	0.2	3.0	8.7	1230	76	13.7	504
		#10061	vehicle	S63845	2.1	0.2	3.1	9.8	1220	29	5.3	288

		#10062	vehicle	S63845	26	41	33	9.3	980	15	139	657
		#10063	vehicle	S63845	32	59	87	9.2	730	15	171	392
		#10064	vehicle	S63845	40	62	76	8.3	990	15	185	641
		#10065	vehicle	S63845	15	16	5.1	10	580	15 fd	fd	fd
										**Mean 15**		
		#10066	tamoxifen	vehicle	0.3	0.1	5.7	11	780	*185*	7.6	1031
		#10067	tamoxifen	vehicle	1.0	0.1	7.4	11	670	*185*	8.1	1049
		#10068	tamoxifen	vehicle	0.5	0.1	7.9	10	640	*185*	9.6	986
		#10069	tamoxifen	vehicle	0.5	0.2	8.4	12	1010	*185*	5.2	1034
		#10070	tamoxifen	vehicle	0.5	0	8.9	11	970	*185*	8.2	933
		#10071	tamoxifen	vehicle	0.6	0.1	8.6	11	830	32	23.6	769
										**Mean >185**		
		#10072	tamoxifen	S63845	1.1	0.1	4.5	9.9	1300	*185*	8.3	1006
		#10073	tamoxifen	S63845	1.5	0.4	4.3	9.8	1160	24	128	265
		#10074	tamoxifen	S63845	1.8	0.1	3.9	9.4	1380	*185*	6.3	1050
		#10075	tamoxifen	S63845	1.9	0.1	5.9	8.8	1390	*185*	5.2	842
		#10076	tamoxifen	S63845	0.9	0.0	5.7	9.6	1240	*185*	9.4	919
		#10077	tamoxifen	S63845	1.4	0.1	3.9	9.6	1250	*185*	6.8	1012
										**Mean >185**		
	**#2232/ #3384** (F) Expt 2	#10100	vehicle	vehicle	2.3	0.7	3.9	11	990	20	68.7	280
		#10101	vehicle	vehicle	4.4	2.5	8.2	10	590	19	120	198
		#10102	vehicle	vehicle	2.5	0.9	8.5	11	860	19	113	301
		#10103	vehicle	vehicle	3.1	1.3	7.9	11	1090	20	127	279
		#10104	vehicle	vehicle	1.9	1.1	7.0	11	940	19	95.5	424
		#10105	vehicle	vehicle	2.8	1.5	10	9.8	730	19	130	307
										**Mean 19**		
		#10106	vehicle	S63845	3.0	0.6	4.3	8.9	2040	20	37.8	483
		#10107	vehicle	S63845	3.4	0.6	3.9	9.5	1610	20	35.7	324
		#10108	vehicle	S63845	5.2	1.3	5.2	9.2	1520	20	162	318
		#10109	vehicle	S63845	4.0	0.5	3.4	9.6	1940	*185*	5.9	1090
		#10110	vehicle	S63845	3.8	0.9	4.3	8.5	1610	20	43.6	351
		#10111	vehicle	S63845	5.2	0.5	4.2	9.2	1790	38	26.6	542
										**Mean 20**		
		#10112	tamoxifen	vehicle	1.1	0.1	4.9	9.5	780	31	113	245
		#10113	tamoxifen	vehicle	1.3	0.1	4.8	9.8	710	30	130	514
		#10114	tamoxifen	vehicle	0.8	0.1	7.0	9.8	740	30	79.4	310
		#10115	tamoxifen	vehicle	1.4	0.1	8.4	8.2	720	29	112	250
		#10116	tamoxifen	vehicle	1.1	0.1	5.9	9.9	810	37	104	307
		#10117	tamoxifen	vehicle	1.3	0.1	6.2	9.9	770	29	23.2	239
										**Mean 30**		
		#10118	tamoxifen	S63845	3.3	0.3	3.7	8.8	1490	31	94.3	227
		#10119	tamoxifen	S63845	3.8	0.4	4.2	9.2	1530	29 fd	fd	fd
		#10120	tamoxifen	S63845	3.1	0.4	5.3	9.6	1450	29	102	394
		#10121	tamoxifen	S63845	3.0	0.2	5.3	8.3	1480	31	113	222

		#10122	tamoxifen	S63845	2.7	0.2	3.7	8.1	1540	31	131	229
		#10123	tamoxifen	S63845	4.3	0.7	4.4	8.4	1530	29	188	297
										**Mean 30**		
	**#2206/ #3427** (F) Expt 2	#10124	vehicle	vehicle	nd	nd	nd	nd	nd	13	342	150
		#10125	vehicle	vehicle	nd	nd	nd	nd	nd	13	295	146
		#10126	vehicle	vehicle	nd	nd	nd	nd	nd	13	171	191
		#10127	vehicle	vehicle	nd	nd	nd	nd	nd	13	333	202
		#10128	vehicle	vehicle	nd	nd	nd	nd	nd	13	343	249
		#10129	vehicle	vehicle	nd	nd	nd	nd	nd	13	272	198
										**Mean 13**		
		#10130	vehicle	S63845	nd	nd	nd	nd	nd	13	458	385
		#10131	vehicle	S63845	nd	nd	nd	nd	nd	13	nd (clot)	nd (clot)
		#10132	vehicle	S63845	nd	nd	nd	nd	nd	13	450	390
		#10133	vehicle	S63845	nd	nd	nd	nd	nd	13	nd (clot)	nd (clot)
		#10134	vehicle	S63845	nd	nd	nd	nd	nd	13	471	347
		#10135	vehicle	S63845	nd	nd	nd	nd	nd	13	464	302
										**Mean 13**		
		#10136	tamoxifen	vehicle	24	28	18	8.4	490	15 fd	nd	nd
		#10137	tamoxifen	vehicle	42	47	43	8.0	240	15	73	87
		#10138	tamoxifen	vehicle	11	15	12	9.5	570	20	197	153
		#10139	tamoxifen	vehicle	37	39	32	8.2	180	15	77	71
		#10140	tamoxifen	vehicle	19	24	16	9.3	580	18	183	211
		#10141	tamoxifen	vehicle	35	42	35	9.0	310	15	54	125
										**Mean 16.5**		
		#10142	tamoxifen	S63845	21	22	15	7.2	780	15 fd	nd	nd
		#10143	tamoxifen	S63845	21	26	16	7.8	1130	16	92	542
		#10144	tamoxifen	S63845	23	27	15	7.9	810	16	144	137
		#10145	tamoxifen	S63845	26	38	18	9.4	1090	19	227	182
		#10146	tamoxifen	S63845	21	24	9.1	7.8	1160	19 fd	nd	nd
		#10147	tamoxifen	S63845	14	14	5.6	7.3	1200	20	185	160
										**Mean 17.5**		

† Identity of mouse with primary (T0) AML which was expanded by transplantation to produce T1 AMLs /identity of mouse with T1 AML used to provide BM cells for drug treatment protocol shown in Fig. 4A.

§Treatment with tamoxifen (200 mg/kg) or its vehicle (peanut oil) by oral gavage was on d3, d4 and d5 after transplantation and iv injection with S63845 (25 mg/kg) or its vehicle (2% Vitamin E in Dulbecco's phosphate-buffered saline) was on d6, d7 d8, d9, d10.

Blood was taken from transplant recipients by retro-orbital bleed on d14 and at point of sacrifice and analysed using Advia 2120. Normal range for WBC, RBC and platelets (PLT) in WEHI's C57BL/6 mice is WBC 7.7±1.2 x10^3 cells/µl, RBC 10.11±0.34 x10^6 cells/µl and PLT 1229±226 x10^9 cells/L respectively. fd = found dead.

Any mice surviving on d195 after treatment were euthanised and autopsied.

Table S5. Impact of combining *Mnt* deletion with BH3 mimetic treatment on survival of mice engrafted with MV4;11

Recipient mouse #	Treatment	Survival (d)	Percentage of hCD45+ cells in BM at day 28 post-transplant	Percentage of hCD45+ cells in BM at autopsy	% hCD45 positive cells in blood at point of death	WBC at autopsy $x10^3$ cells/µl[1]	RBC at autopsy $x10^6$ cells/µl[1]	PLT at autopsy $x10^9$ cells/L[1]
194	DOX only	39	9.89%	31.70%	2.88%	2.2	9.31	724
195	DOX only	36	32.90%	42.20%	42.70%	4.65	9.02	556
196	DOX only	34	30.90%	41.00%	40.70%	4.68	8.6	599
197	DOX only	39	11.30%	29.70%	56.00%	8.56	7.95	566
198	DOX only	36	14.40%	24.70%	10.40%	3.65	8.84	661
199	DOX only	34	31.90%	37.30%	44.10%	3.8	8.83	658
200	VEN only	33	31.80%	41.90%	81.90%	42.04	6.58	384
201	VEN only	32	17.30%	23.60%	50.30%	5.87	8.16	606
202	VEN only	32	40.00%	49.70%	63.20%	6.24	7.98	560
203	VEN only	34	26.80%	42.50%	22.90%	3.02	8.14	634
204	VEN only	35	13.90%	34.60%	29.00%	5.92	8.31	409
205	VEN only	33	16.60%	38.80%	33.40%	6.96	8.66	638
206	DOX + VEN	39	17.30%	48.90%	47.60%	3.39	8.44	626
207	DOX + VEN	39	0.0485	45.90%	46.30%	6.43	6.94	445
208	DOX + VEN	42	0.131	39.10%	78.50%	19.81	7.79	514
209	DOX + VEN	36	0.273	41.40%	23.50%	4.05	6.73	707
210	DOX + VEN	39	0.0896	49.50%	56.10%	8.31	7.67	583
211	DOX + VEN	39	0.177	43.20%	66.90%	25.7	3.54	330
212	Untreated	26	No data	44.80%	40.90%	10.8	8.03	447
213	Untreated	26	No data	46.20%	45.20%	5.9	8.76	589
214	Untreated	28	50.20%	47.00%	58.40%	5.9	8.46	599
215	Untreated	28	41.60%	38.20%	39.50%	7.1	8.53	522
216	Untreated	27	No data	42.30%	27.00%	6.95	8.42	536
217	Untreated	28	50.50%	47.60%	46.10%	6.1	7.62	535

[1]Blood was taken by cardiac bleed at point of euthanasia and analysed using the Mindray BC-5000. Normal range for WBC, RBC and platelets (PLT) in NSG mice is WBC 1.23± 0.5 $x10^3$ cells/µl, RBC 8.27±0.38 $x10^6$ cells/µl and PLT 974±93 $x10^9$ cells/L respectively (Ref. Layssol-Lamour CJ, Sarry JE, Braun JD, Trumel C, Bourgès-Abella NH. Reference Values for Hematology, Plasma Biochemistry, Bone Marrow Cytology and Bone Histology of NOD.*Cg-Prkdcscid Il2rgtm1Wjl/* SzJ Immunodeficient Mice. J Am Assoc Lab Anim Sci. 2021 Jan 1;60(1):4-17. doi: 10.30802/AALAS-JAALAS-20-000020. Epub 2020 Oct 12. PMID: 33046180; PMCID: PMC7831346).

MNT: a new target for AML

Karla C Fischer[1,2], Veronique Litalien[1], Sarah T Diepstraten[1,2], Michelle Jahja[1,2], Fiona C Brown[1,2], Gemma L Kelly[1,2], Andrew H Wei[1,2,3,4] and Suzanne Cory[1,2]

[1]The Walter and Eliza Hall Institute of Medical Research, Melbourne, Victoria, Australia; [2]Department of Medical Biology, The University of Melbourne, Melbourne, Victoria, Australia; [3]Department of Haematology, Peter MacCallum Cancer Centre, Melbourne, Victoria, Australia, [4]Royal Melbourne Hospital, Melbourne, Victoria, Australia

Supplemental Materials and methods

Mouse breeding

All mice used in this study were maintained on a C57BL/6 background. They were bred, housed and monitored in the Bioservices Facility at the Walter and Eliza Hall Institute (WEHI) under the supervision of trained veterinarians and in accordance with WEHI animal ethics committee regulations and the Australian Code for the Care and Use of Animals for Scientific purposes. $Mnt^{fl/fl}$ and $Mnt^{fl/fl}/CreERT2$ genotypes were bred using $Mnt^{fl/+}$[1] and $Rosa26^{CreERT2/+}$[2] (hereafter *CreERT2*) mice. The ERT2 moiety of the CRE-ERT2 protein retains the recombinase in the cytoplasm until tamoxifen administration *in vivo* or 4-hydroxy tamoxifen (4-OHT) treatment *in vitro* releases this inhibition, permitting its entry into the nucleus and recombination of genomic *loxP* sites[3]. Note that homozygous *Mnt* deletion, initially reported as perinatal lethal[1], is fatal at embryonic day ~E10 in C57BL/6 mice bred at WEHI[4].

Mouse genotype analysis

Mouse genotypes were determined by PCR analysis of tail DNA isolated by standard procedures. 1 µL of tail DNA extract was added to 19 µL GoTaq Green Master Mix (Promega M7123) containing the primers (final concentration 0.5 pmol/µL). The PCR program used was 94º C for 3 minutes followed by 30 cycles (94 ºC for 30 seconds, 58 ºC for 30 seconds, 72 ºC for 40 seconds) and finally 72 ºC for 5 minutes. PCR products were separated by gel electrophoresis on a 2% DNA grade agarose gel (Bioline BIO-41025) in TAE buffer (40 mM

Tris Acetate, 1 mM EDTA pH 8.0) containing ethidium bromide (0.2 µg/ml final concentration from Sigma) and imaged using a Gel DOCTM XR+Gel Documentation system (Bio-Rad). PCR oligonucleotide primers used were:

***FloxMnt*:** wt 178 bp, ko 579 bp[1]
Mnt CKO-2: 5'-GTCTCAAGTCGTGGGCATTG-3'
Hygro R-S: 5'-GATGTAGGAGGGCGTGGATA-3'
Hygro R-R: 5'-GATGTTGGCGACCTCGTATT-3'
Mnt Seq1: 5'-CAGATTCAGTGTCCCCTGCT-3'

***DelMnt*:** ko 386 bp, wt 147 bp[1]
Mnt ko3: 5'-CAGGTCCTCCAAAAGAGCAG-3'
Mnt ko4: 5'-GGAGCAATGTGGAGAGAAGC-3'
Mnt wt-sense: 5'-CAGTCCCTGAAGAGGAAGGA-3'
Mnt wt-rev2: 5'-CCGGAGCACACGATCTATCT-3'

RosaCre: rosa 700 bp, cre 450 bp
RosaS se: 5'-GCCAATGCTCTGTCTAGGGGTTGG-3'
RosaS as: 5'-CTTGCTCTCCCAAAGTCGCTCTGAG-3'
RosaS cre as: 5'-TCGTTGCATCGACCGGTAATGCAGGC-3'

Analysis of mice with *CreERT2*-mediated *Mnt* deletion

8-week-old *CreERT2, Mnt$^{fl/fl}$* and *Mnt$^{fl/fl}$ CreERT2* mice were treated with tamoxifen (T5648, Sigma-Aldrich) on 3 consecutive days by oral gavage (200 mg/kg body weight). Mice were closely monitored for any signs of illness post tamoxifen treatment, such as weight loss, anaemia, lethargy and irregular breathing. Whole blood obtained from mice by retro-orbital bleed was analysed using the Avida 2120 haematological analyser (Bayer). At autopsy, blood, bone marrow (femurs, tibiae) and organs (sternum, thymus, lung, spleen, liver and kidneys) were collected for analysis. Single cell suspensions from bone marrow were obtained using a mortar and pestle to crush the bones and a 40 µM cell strainer. RBC were lysed with NH_4Cl buffer (156 mM NH_4Cl, 11.9 mM $NaHCO_3$, 0.097 mM EDTA).

Cellular composition of haemopoietic tissues was determined by flow cytometry: single cell suspensions of bone marrow or spleen were prepared in FACS buffer (phosphate-buffered saline (PBS) supplemented with 1% heat inactivated foetal bovine serum (HI-FBS) (A4503,

Sigma-Aldrich)) and cells were counted using the Bio-Rad TC20 automated cell counter (Bio-Rad Laboratories, Inc., Hercules, California, United States). Cells were incubated in FACS buffer on ice for 2 hours with the fluorochrome-conjugated monoclonal antibodies listed below.

Haemopoietic stem/progenitor cells were analysed by cell surface staining with the following antibodies: CD117(c-Kit)-APC (clone 2B8, BD, Biosciences, San Jose, CA, USA); SCA-1-A594 (clone E13, in-house); CD48-FTIC (clone HM48-1, eBioscience); CD150-BV421 (clone TC15-12F12.2, Biolegend, San Diego, CA, USA); CD41-PECy7 (clone MWReg30, Biolegend, San Diego, CA, USA); CD16/32-PerCPCy5.5 (clone 2.4G2, BD, Biosciences, San Jose, CA, USA); CD105-PE (clone MJ718, eBioscience) and lineage exclusion markers CD4-A700 (clone GK1.5); CD8-A700 (clone 53.6.7); GR-1-A700 (clone RB6-8C5); F4/80-A700; CD19-A700 (clone 1D3); B220 (CD45R)-A700 (clone RA3-6B2); Ly6G-A700 (clone1A8); TER119-A700 (clone Ter-119); NK1.1-A700 (clone PK136) (produced and labelled in-house).

Mature haemopoietic lineages were analysed by cell surface staining with the following antibodies: B220 (CD45R)-APC-Cy7 (clone RA3-6B2, BD, Biosciences, San Jose, CA, USA), Gr-1-APC (clone RB6-8C5, in-house), CD11b (Mac-1)-V450 (clone M1/70, BD, Biosciences, San Jose, CA, USA), CD3-FITC (clone 17A2, in-house), NK1.1-PE-Cy7 (clone PK136, Biolegend, San Diego, CA, USA), TER119-PE (clone Ter-119, in-house), CD19-PerCP-Cy5.5 (clone 1D3, BD, Biosciences, San Jose, CA, USA).

Flow cytometric analysis was performed on the Fortessa1 instrument (BD Biosciences, San Jose, CA, USA; with BD FACS Diva software) and data were analysed using FlowJo analysis software (v10.5.3; FlowJo Enterprise). Dead cells were excluded from analysis by staining with the viability dye Hydroxystilbamidine (Fluoro-Gold; Sigma-Aldrich #39286-10MG-F) (8μg/ml) and doublets were gated out by SSC-W/SSC-H and FSC-W/FSC-H exclusion.

Analysis of mice with AML

Sick mice were humanely euthanised, autopsied and tissues collected for histology, immunophenotyping, PCR and western blot analysis. Peripheral blood was analysed using an ADVIA haematology analyser (Bayer). Soft tissues (spleen, liver, kidneys, lung) and sternum were collected into 10% formalin before embedding in paraffin wax and staining with haematoxylin and eosin (H&E) for histological analysis. Cellular composition of bone marrow and spleen was determined by flow cytometry. Single cell suspensions were incubated with the following fluorochrome-conjugated antibodies Gr-1-APC (clone RB6-8C5, in-house); CD11b (Mac-1)-V450 (clone M1/70, BD, Biosciences, San Jose, CA, USA); CD45.1 (Ly5.1)-PE-Cy7

(clone A20.1; BD, Biosciences, San Jose, CA, USA); CD45.2 (Ly5.2)-PE (clone S450-15.2, in-house)) and Fluoro-Gold as previously described. Samples were analysed on a Fortessa 1 flow cytometer (BD Biosciences, San Jose, CA, USA; with BD FACS Diva software). Dead, Fluoro-Gold positive, cells were excluded from analysis and cell doublets were gated out by SSC-W/SSC-H and FSC-W/FSC-H exclusion using FlowJo software (v10.5.3; FlowJo Enterprise).

***Mnt* deletion and treatment of *MLL::AF*9 AML cell lines with BH3 mimetic drugs**

Cells were plated into 96-well flat-bottom plates in triplicate at $5x10^4$ cells/well and incubated with vehicle (ethanol) or 4-OHT (Sigma Cat#H7904) at a final concentration of 0.5 µM for 24 hours. Where indicated, pan-caspase inhibitor Q-VD-OPh (MedChem Express, # HY-12305) was added at 25 µM 30 minutes prior to treatment with 4-OHT. The following BH3 mimetic drugs were used: S63845 (MCL-1 inhibitor; Active Biochem, #A-6044), ABT-199 (BCL-2 inhibitor; Active Biochem #A-1231) and A-1331852 ($BCL-X_L$ inhibitor; AbbVie, gift from Dr G. Lessene, WEHI) all dissolved in DMSO. After treatment for 48 hours, cells were transferred into a V-bottom 96-well plate, spun at 500 x g for 5 minutes and resuspended in Annexin V binding buffer (10 mM HEPES pH 7.4, 140 mM NaCl, 2.5 mM $CaCl_2$) containing Annexin V-Alexa Fluor 647 (1:1000, made in house) and propidium iodide (PI; 1 µg/mL; Sigma-Aldrich, #P4170). Live cells (Annexin V^-PI^-) were quantified on an LSR II flow cytometer (BD Biosciences). Data analysis was performed using FlowJo v10 and GraphPad Prism v9.

S63845 treatment of transplanted AMLs after *Mnt* deletion

Insufficient primary (T0) bone marrow cells were available for this experiment. Therefore, T0 AMLs (C57BL/6-Ly5.2) were expanded by injecting cryopreserved T0 spleen cells via the tail vein into multiple C57BL/6-Ly5.1 mice (2×10^6 cells/mouse) and cryopreserving bone marrow cells of sick recipients (designated T1). For treatment studies of T1 AMLs, 24 non-irradiated mice (8-wk-old C57BL/6-Ly5.1) were injected IV on day 0 with 0.5×10^6 bone marrow cells from one T1 mouse and drug regimens commenced 3 days later. On day 3, 4 and 5, half of the cohort was treated by oral gavage with tamoxifen (200 mg/kg body weight; Sigma-Aldrich #T5648), the other half with an equal volume of vehicle (peanut oil). Then each treatment group was split again, and half of the mice were injected via the tail vein with S63845 (25 mg/kg body weight; Active Biochem; #6044), the other half with vehicle alone (2% Vitamin E (D-alpha-tocopherol polyethylene glycol 1000 succinate, BioXtra, water soluble vitamin E conjugate, Sigma #57668) in Dulbecco's phosphate-buffered saline) on days 6-10. Mice were

monitored daily for symptoms of disease onset and euthanised humanely when showing signs of AML-induced stress, significant weight loss (>10% of initial body weight), or at the experimental end point (195 days).

Western blot analysis

Cell pellets were washed in cold phosphate-buffered saline (PBS) and total cell lysates were prepared by lysing directly into SDS PAGE gel loading buffer (50 mM Tris-HCl, 2% SDS, 0.04% bromophenol blue, 10% glycerol, 5% 2-mercaptoethanol) at a cell density of 1.5 x 10^4 cells/µL of buffer. Lysates were denatured at 95ºC for 10 minutes and treated with Benzonase (50U per 100µL lysate; Novagen Cat#71206) for 15 minutes at 37ºC followed by a heat-inactivation step at 95 ºC for 2 minutes to digest DNA prior to resolving the samples by SDS-PAGE electrophoresis in Tris/glycine/SDS running buffer (Bio-Rad # #1610732) using 12% Mini-PROTEAN® TGX™ Precast Protein Gels (BioRad #4561045 or #4561045) or 4-15% Mini-PROTEAN® TGX™ Precast Protein Gels (BioRad #4561085). Molecular weights were estimated using the BenchMark™ Pre-stained Protein Ladder (Invitrogen Cat#10748010) or Precision Plus Protein Dual Color Standard (Bio Rad #1610374). Proteins separated by SDS-PAGE were transferred on to PVDF membranes (Invitrogen Cat# IB24001) using an iBlot2 gel transfer device (Invitrogen Cat#IB21001). Membranes were blocked with 5% non-fat dry milk powder dissolved in Tris Buffered Saline with 0.1% Tween 20 (TBS-T) containing 0.1% Tween 20 (TBS-T) (Sigma Cat#P1379), then incubated overnight at 4º C with primary antibodies (see below) in blocking solution (5% non-fat dry milk powder in 1x TBS-T or 5% BSA (Sigma #A3983) in 1xTBS-T). Following washes in TBS-T (3x10 minutes at room temperature), membranes were incubated with the appropriate horseradish peroxidise (HRP)-conjugated secondary antibody (anti-Rabbit-IgG, anti-Mouse-IgG or anti-rat-IgG antibodies conjugated to HRP) at room temperature for 60 min, followed by washes in TBS-T (3x10 minutes at room temperature). Protein bands were visualised using Immobilon Forte Western HRP substrate (Millipore Cat#WBLUF0100) and imaged on the ChemiDoc TM Touch Imaging System (Bio-Rad Cat#1708370). Prior to re-probing with another antibody, membranes were stripped of secondary antibody by incubating the membranes for 5 min at room temperature with sodium hydroxide stripping buffer (0.5M NaOH) followed by 3x10 minute washes in TBS-T.

Primary antibodies used were:

MNT (1:1000, Bethyl Cat#A303-626A); MLL1-N (1:1000, clone D2M7U, Cell Signalling Cat#14689S); human BCL-2 (1:1000, clone BCL-2-100; WEHI mAb Facility); mouse BCL-2

(1:1000, clone 3F11; WEHI mAb Facility); BCL-X_L (1:5000, clone E18, Abcam #ab32370); BIM (1:1000, Enzo Life Sciences #ADI-AAP-330-E); ACTIN-HRP (1:5000, clone C4, Santa Cruz #sc-47778); c-MYC (1:1000, clone D84C12, Cell Signalling Cat#5605S); MCL-1 (1:1000, clone 19C4-15, WEHI mAB Facility), PARP1 (1:1000, clone EPR18461, Abcam, #ab191217); BAX (1:2000, clone #49F9-13-3, WEHI mAb Facility); BAK (1:5000, clone DF9, Sigma #5987).

Secondary antibodies used were anti-rabbit IgG-HRP (1:5000, Southern Biotech Cat#4010-05); anti-mouse IgG-HRP (1:2000, Jackson ImmunoResearch Cat#115-035-071); anti-rat IgG-HRP (1:5000, Southern Biotech Cat#3010-05); anti-hamster IgG-HRP (1:5000, Southern Biotech Cat#6060-05).

Uncropped blots are shown in the supplementary "original data" file.

Human AML cell lines

Human *MLL*-fusion gene AML cell lines MV4;11[5] and THP-1[6] were originally imported to WEHI from ATCC (American Type Culture Collection;VI USA), and MOLM-13[7] and OCI-AML3[8] from DSMZ (Deutsche Sammlung von Mikroorganismen unde Zellkulturen, Leibniz, Germany). MV4;11, MOLM-13 and THP-1 cell lines were cultured in RPMI-1640 medium (Gibco #31800089) supplemented with 10% heat-inactivated FCS, 100 U/ml penicillin and 100 µg/mL streptomycin. The OCI-AML3 cell line was cultured in MEM alpha medium with GlutaMAX™ supplement (Gibco #32561037) supplemented with 10% heat-inactivated FCS (Sigma #F9423). All human leukaemia cell lines were maintained at 37ºC with 5% CO_2 for <2 months, authenticated by STR profiling at CellBank Australia (CMRI) and regularly tested negative for mycoplasma (Lonza MycoAlert).

CRISPR/Cas9 deletion of *MNT* in human AML cell lines

CRISPR/Cas9 gene editing was employed to generate *MNT* KO human AML cell lines, using lentiviral vectors developed by Aubrey et al[9]. The first vector confers constitutive expression of Cas9 and mCherry, linked via the self-cleaving T2A peptide, and cells expressing high levels of Cas9 are selected by sorting for mCherry^{hi} cells. The second vector encodes a doxycyclin-inducible sgRNA cassette, driven by the H1 promoter containing a Tet-operator site (tetO), linked to another cassette in which a ubiquitin promoter (Ubi-P) drives constitutive expression of a tetracycline repressor (TetR) linked via the T2A peptide to the enhanced Green

Fluorescence Protein (eGFP) (FgH1tUTG backbone (Addgene Plasmid #70183). Two independent sgRNAs (*sghMNT*#2 5'-CGCATGGAGGCGCCACCCCT-3' and *sghMNT*#4 5'-CCGGACTCAGCATTAAGGAG-3' abbreviated *MNT#2* and *MNT#4* respectively) directed against exon 2 of human *MNT* (M. Michla and H.V. Nguyen, unpublished), as well as a non-targeting control sgRNA directed against mouse *Bim* (*sgmBim3* 5'-GCACAGGAGCTGCGGCGGAT-3', abbreviated m*Bim*)[9] were cloned into the second vector. Transduced cells are eGFP-positive but do not express the sgRNA until exposed to doxycycline, which binds to the Tet repressor, releasing it from the Tet Operator.

HEK 293T cells were transfected with vector DNA and packaging plasmids pMDL, pRSV-REV and VSVg via the standard calcium precipitation procedure[9] and lentivirus-containing supernatants were collected after 48 hours, and passed through a 0.45 mm filter prior to infection of human AML cell lines by centrifugation at 2,200 rpm for 2 h at 32° C in the presence of polybrene (final concentration 8 μg/mL). Cells were incubated overnight at 37^0 C, then the virus-containing medium was removed and replaced with fresh medium. After >72 hours at 37^0C, mCherry^{hi}GFP^{hi} cells were sorted on a FACSAria Fusion flow cytometer (BD Biosciences) and the pooled population was frozen in DMSO and stored at -80°C. To induce sgRNA expression, pools were treated for 5 days with doxycycline hyclate (Sigma, #D9891) at 1 μg/mL, adding fresh doxycycline every second day.

Human AML xenograft mouse model

All mouse studies were conducted with approval from the WEHI animal ethics committee in accordance with the WEHI animal ethics committee regulations and the Australian Code for the Care and Use of Animals for Scientific purposes. For *in vivo* studies, the BCL-2 inhibitor ABT-199/venetoclax (MedChemExpress, #MCE-HY-15531) was dissolved in 5% DMSO (Sigma-Aldrich, cat#D5879) + 50% PEG 300 (Sigma-Aldrich, cat#202371), 5% Tween 80 (Sigma-Aldrich, cat#P8192) and 40% distilled water. NOD-SCID IL2Rγ-null (NSG) mice (8-10 weeks-old) were obtained from the WEHI breeding facility and injected IV with 5×10^5 MV4;11 human cells expressing Cas9 and two separate doxycycline-inducible single guide (sg) RNAs (sgh*MNT#2, sghMNT*#4) both targeting human *MNT.* Doxycycline treatment (600 mg/kg doxycycline hyclate in chow, fed orally ad libitum; Specialty Feeds #SF08-026) commenced on day 4 post transplantation until the end of the experiment. Venetoclax was delivered by oral gavage every weekday (200 μL 50 mg/kg body weight) for 4 weeks. Mice were monitored daily for symptoms of disease onset and euthanised humanely when showing

signs of AML-induced stress. Tumour burden was assessed by flow cytometric analysis of human CD45$^+$ cells in bone marrow isolated by intrafemoral sampling on day 28 post transplantation, and in bone marrow isolated from flushed femurs at end point.

Leukaemia burden was determined by flow cytometric assessment of bone marrow by gating on live human CD45$^+$ (hCD45-APC-Cy7) cells. Cell viability was assessed by LIVE/DEAD Fixable Yellow (ThermoFisher Scientific #L34959) and cells were fixed with eBioscience Foxp3/Transcript Factor Staining Buffer Set (Invitrogen #00-5523-00) before analysis on a Fortessa1analyser.

Antibodies used for flow cytometry

Antibody specificity	Fluoro-chrome	Clone	Source	Catalogue #	Dilution
CD117(c-Kit)	APC	2B8	BD Biosciences	561074	1:200
SCA-1 (Ly-6A/E)	A594	E13	WEHI mAb Lab	N/A	1:200
CD48	FITC	HM48-1	eBioscience	11-0481-82	1:300
CD150	BV421	TC15-12F12.2	BioLegend	115926	1:200
CD41	PECy7	MWReg30	BioLegend	133916	1:300
CD16/32	PerCPCy5.5	2.4G2	BD Biosciences	560540	1:200
CD105	PE	MJ718	eBioscience	12-1051-82	1:200
CD4	A700	GK1.5	WEHI mAb Lab	N/A	1:400
CD8	A700	53.6.7	WEHI mAb Lab	N/A	1:400
Gr-1	A700	RB6-8C5	WEHI mAb Lab	N/A	1:400
F4/80-A700	A700	F4/80	WEHI mAb Lab	N/A	1:400
CD19	A700	1D3	WEHI mAb Lab	N/A	1:400
B220 (CD45R)	A700	RA3-6B2	WEHI mAb Lab	N/A	1:400
Ly6G	A700	1A8	WEHI mAb Lab	N/A	1:400
TER119	A700	Ter-119	WEHI mAb Lab	N/A	1:400
NK1.1	A700	PK136	WEHI mAb Lab	N/A	1:400

B220 (CD45R)	APC-Cy7	RA3-6B2	BD Biosciences	561102	1:200
Gr-1	APC	RB6-8C5	WEHI mAb Lab	N/A	1:50
CD11b (Mac-1)	V450	M1/70	BD Biosciences	560455	1:250
CD3	FITC	17A2	WEHI mAb Lab	N/A	1:50
NK1.1	PE-Cy7	PK136	BioLegend	108714	1:200
TER119	PE	Ter-119	WEHI mAb Lab	N/A	1:50
CD19	PerCP-Cy5.5	1D3	BD Biosciences	561113	1:200
Gr-1	APC	RB6-8C5	WEHI mAb Lab	N/A	1:50
mCD45.1 (Ly5.1)	PECy7	A20.1	BD Biosciences	561872	1:300
mCD45.2 (Ly5.2)	PE	S450-15.2	WEHI mAb Lab	N/A	1:400
hCD45	APC-Cy7	HI30	BioLegend	304014	1:1000
mCD45	BV711	30-F11	BD Biosciences	563709	1:100

Antibodies used for Western blotting

**Target**	**Clone**	**Dilution**	**Species raised in**	**Source**
**Primary antibodies**				
MNT	polyclonal	1:1000	Rabbit	Bethyl Cat#A303-626A
MLL1-N	D2M7U	1:1000	Rabbit monoclonal	Cell Signalling #1469S
c-MYC	D84C12	1:1000	Rabbit	Cell Signalling Cat#5605S
MCL-1	19C4-15	1:2000	Rat	WEHI mAb Facility
BCL-2	clone 3F11	1:1000	Hamster	WEHI mAb Facility
BCL-XL	E18	1:5000	Rabbit	Abcam #ab32370
BIM	polyclonal	1:1000	Rabbit	Enzo Life Sciences, #ADI-AAP-330-E
PARP1	EPR18461	1:1000	Rabbit	Abcam, #ab191217
BAK	DF9	1:5000	Rabbit	Sigma#5897

BAX	49F9-13-3	1:2000	Rat	WEHI mAb Facility
β-ACTIN-HRP	C4	1:5000	Mouse	Santa Cruz #sc-47778
**Secondary antibodies**				
Mouse IgG	N/A	1:2000	Goat	Southern Biotech, #1010-05
Rat IgG	N/A	1:5000	Goat	Southern Biotech, #3010-05
Rabbit IgG	N/A	1:5000	Goat	Southern Biotech, #4010-05
Hamster IgG	N/A	1:5000	Goat	Southern Biotech, #6060-05

References

1. Toyo-oka K, Hirotsune S, Gambello MJ, Zhou ZQ, Olson L, Rosenfeld MG*, et al.* Loss of the Max-interacting protein Mnt in mice results in decreased viability, defective embryonic growth and craniofacial defects: relevance to Miller-Dieker syndrome. *Hum Mol Genet* 2004, **13**(10)**:** 1057-1067.
2. Seibler J, Zevnik B, Kuter-Luks B, Andreas S, Kern H, Hennek T*, et al.* Rapid generation of inducible mouse mutants. *Nucleic Acids Res* 2003, **31**(4)**:** e12.
3. Feil R, Brocard J, Mascrez B, LeMeur M, Metzger D, Chambon P. Ligand-activated site-specific recombination in mice. *Proc Natl Acad Sci U S A* 1996, **93**(20)**:** 10887-10890.
4. Nguyen HV, Vandenberg CJ, Ng AP, Robati MR, Anstee NS, Rimes J*, et al.* Development and survival of MYC-driven lymphomas require the MYC antagonist MNT to curb MYC-induced apoptosis. *Blood* 2020, **135**(13)**:** 1019-1031.
5. Lange B, Valtieri M, Santoli D, Caracciolo D, Mavilio F, Gemperlein I*, et al.* Growth factor requirements of childhood acute leukemia: establishment of GM-CSF-dependent cell lines. *Blood* 1987, **70**(1)**:** 192-199.
6. Tsuchiya S, Yamabe M, Yamaguchi Y, Kobayashi Y, Konno T, Tada K. Establishment and characterization of a human acute monocytic leukemia cell line (THP-1). *Int J Cancer* 1980, **26**(2)**:** 171-176.
7. Matsuo Y, MacLeod RA, Uphoff CC, Drexler HG, Nishizaki C, Katayama Y*, et al.* Two acute monocytic leukemia (AML-M5a) cell lines (MOLM-13 and MOLM-14)

with interclonal phenotypic heterogeneity showing MLL-AF9 fusion resulting from an occult chromosome insertion, ins(11;9)(q23;p22p23). *Leukemia* 1997, **11**(9)**:** 1469-1477.

8. Quentmeier H, Martelli MP, Dirks WG, Bolli N, Liso A, Macleod RA*, et al.* Cell line OCI/AML3 bears exon-12 NPM gene mutation-A and cytoplasmic expression of nucleophosmin. *Leukemia* 2005, **19**(10)**:** 1760-1767.

9. Aubrey BJ, Kelly GL, Kueh AJ, Brennan MS, O'Connor L, Milla L*, et al.* An inducible lentiviral guide RNA platform enables the identification of tumor-essential genes and tumor-promoting mutations in vivo. *Cell Rep* 2015, **10**(8)**:** 1422-1432.
